# New strategies to enhance the efficiency and precision of drug discovery

**DOI:** 10.3389/fphar.2025.1550158

**Published:** 2025-02-11

**Authors:** Qi An, Liang Huang, Chuan Wang, Dongmei Wang, Yalan Tu

**Affiliations:** Scientific Research and Teaching Department, Public Health Clinical Center of Chengdu, Chengdu, Sichuan, China

**Keywords:** drug discovery, click chemistry, targeted protein degradation (TPD), DNA-encoded libraries (DELs), computer-aided drug design (CADD)

## Abstract

Drug discovery plays a crucial role in medicinal chemistry, serving as the cornerstone for developing new treatments to address a wide range of diseases. This review emphasizes the significance of advanced strategies, such as Click Chemistry, Targeted Protein Degradation (TPD), DNA-Encoded Libraries (DELs), and Computer-Aided Drug Design (CADD), in boosting the drug discovery process. Click Chemistry streamlines the synthesis of diverse compound libraries, facilitating efficient hit discovery and lead optimization. TPD harnesses natural degradation pathways to target previously undruggable proteins, while DELs enable high-throughput screening of millions of compounds. CADD employs computational methods to refine candidate selection and reduce resource expenditure. To demonstrate the utility of these methodologies, we highlight exemplary small molecules discovered in the past decade, along with a summary of marketed drugs and investigational new drugs that exemplify their clinical impact. These examples illustrate how these techniques directly contribute to advancing medicinal chemistry from the bench to bedside. Looking ahead, Artificial Intelligence (AI) technologies and interdisciplinary collaboration are poised to address the growing complexity of drug discovery. By fostering a deeper understanding of these transformative strategies, this review aims to inspire innovative research directions and further advance the field of medicinal chemistry.

## Graphical Abstract

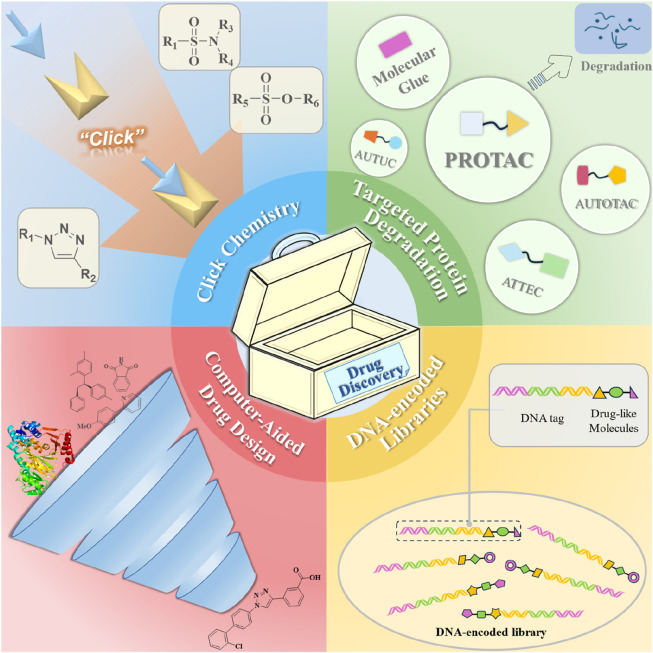

## 1 Introduction

Medicinal chemistry is the interdisciplinary field that focuses on the design, development, and optimization of pharmaceutical compounds. It combines principles from chemistry, biology, and pharmacology to create bioactive molecules that can effectively treat diseases (Francesco, 2023). Key research contents include the synthesis of new compounds, structure-activity relationship (SAR) studies, optimization of drug properties (such as solubility and bioavailability), and the evaluation of pharmacological effects. The discovery and design of small molecule drugs have been pivotal in the field of medicinal chemistry, serving as the cornerstone for developing new therapeutics to combat a myriad of diseases (Campbell et al., 2018). Small molecules have historically played a critical role in the development of effective treatments for a wide range of diseases, including cancer, infectious diseases, and chronic illnesses (Zhong et al., 2021; Davis et al., 2020; Parra Sánchez et al., 2022). Despite their significance, the process of small molecule drug discovery is fraught with challenges, such as high attrition rates in clinical trials, the complexity of biological systems, and the need for precision-targeted therapies (Zhong et al., 2021; Keserü and Makara, 2009; Bedard et al., 2020). As such, there is a pressing need for innovative approaches that can enhance the efficiency and success of small molecule drug design.

One of the primary challenges in the drug discovery process is the vast chemical space that needs to be navigated (Saldívar-González and Medina-Franco, 2022). Traditional methods often rely on trial-and-error approaches, which are not only time-consuming but also resource-intensive. Additionally, the complexity of biological systems and the diverse nature of diseases necessitate a more targeted and strategic approach to drug design. In this context, several new methodologies have emerged, promising to transform the landscape of small molecule drug discovery.

Click Chemistry has garnered significant attention as a powerful tool in drug discovery. It enables the rapid synthesis of diverse compound libraries through highly efficient and selective reactions (Zhao et al., 2024; Wilson Lucas et al., 2023). Click chemistry’s modular nature allows for the straightforward incorporation of various functional groups, facilitating the optimization of lead compounds and enabling the creation of complex structures from simple precursors (Jiang et al., 2019). This approach not only accelerates the discovery process but also enhances the likelihood of identifying compounds with desirable pharmacological properties.

Targeted Protein Degradation (TPD) strategies represent another groundbreaking advancement in small molecule drug design (Wells and Kumru, 2024). Unlike traditional inhibitors that aim to block protein activity, TPD technologies employ small molecules to tag undruggable proteins for degradation via the ubiquitin-proteasome system or autophagic-lysosomal system (Song et al., 2023). This novel approach provides a means to address undruggable targets and offers a new therapeutic paradigm for conditions where conventional small molecules have fallen short. By harnessing the body’s own degradation machinery, TPD strategies enable the selective removal of disease-associated proteins, thereby providing an innovative pathway for drug discovery.

DNA-Encoded Libraries (DELs) have emerged as a widely used technology that allows for the high-throughput screening of vast chemical libraries (Peterson and Liu, 2023). DELs utilize DNA as a unique identifier for each compound, facilitating the simultaneous testing of millions of small molecules against biological targets (Dockerill and Winssinger, 2023). This technology not only streamlines the identification of potential drug candidates but also allows for the exploration of chemical diversity in an unprecedented manner. The ability to rapidly probe the interaction of small molecules with target proteins enhances the efficiency of lead discovery and optimization, making DELs an essential tool in modern drug development.

Computer-Aided Drug Design (CADD) has also significantly influenced the drug discovery landscape by employing computational methods to predict the binding affinity of small molecules and specific targets (Sadybekov and Katritch, 2023; Jiménez-Luna et al., 2021) This approach significantly reduces the time and resources required for experimental screening (Giordano et al., 2022). With advancements in artificial intelligence (AI), CADD is becoming increasingly sophisticated, enabling researchers to simulate complex biological interactions and refine drug design more effectively (You et al., 2022). This predictive capability not only accelerates the discovery process but also enhances the precision of drug design, addressing the critical need for tailored therapies.

Each approach brings unique advantages, addressing specific challenges in the drug development pipeline and paving the way for innovative therapeutic solutions. The integration of these technologies promises to enhance the efficiency of drug discovery, reduce attrition rates in clinical trials, and ultimately lead to the development of more effective and targeted therapies. This review aims to provide a comprehensive overview of recent advancements in these approaches, to explore their underlying principles, and their potential impact on the future of medicinal chemistry. To illustrate the utility of these techniques, we highlight exemplary small molecules developed over the past decade, emphasizing their role in accelerating drug development. Furthermore, we summarize representative marketed drugs and investigational new drugs, illustrating these technologies' direct contributions to clinical practice. Finally, we discuss the future prospects and challenges of each approach. By fostering an understanding of these emerging strategies, we aim to inspire new research directions and collaborations that will further advance the field of medicinal chemistry.

## 2 Click chemistry

Click chemistry, a combinatorial methodology first introduced by Professor Sharpless in 2001, revolutionized the rapid synthesis of C-X-C atom frameworks (Kolb et al., 2001). This approach has since become an essential tool in the medicinal chemist’s toolbox, offering significant advantages such as broad substrate scope, high yield, stereospecificity, operational simplicity, and the formation of only benign by-products that can often be removed without chromatography (Caselli et al., 2015; Le Droumaguet et al., 2022). Additionally, click reactions are conducted using environmentally benign solvents, further enhancing their utility in drug discovery (Shankaraiah et al., 2019). In contrast to traditional drug design, click chemistry employs modular reactions to efficiently create new drug-like molecules. Moreover, these reactions serve as versatile linkers for small molecules and proteins. An example is their application in the synthesis of proteolysis targeting chimeras (PROTACs), where click chemistry links two pharmacophores via a specific scaffold to produce compounds with high target affinity (Wurz et al., 2017). Click chemistry is also instrumental in constructing diverse compound libraries, offering a robust platform for screening bioactive molecules and potential therapeutic agents. Importantly, target-templated *in situ* click chemistry allows for the direct generation of hits within the binding pocket of a target, streamlining the discovery of enzyme inhibitors and other bioactive compounds. This powerful method significantly accelerates both the synthesis and screening processes in drug discovery (Kugler et al., 2023; Glassford et al., 2016).

### 2.1 The conventional click chemistry

Conventional click chemistry has found widespread application in various domains of drug discovery, facilitating the modular synthesis of new drug-like molecules, serving as a linker for PROTACs or pharmacophore groups, and enabling the rapid construction of compound libraries. This efficient, chemo-selective synthesis method allows for the coupling of molecular fragments under mild reaction conditions, making it highly desirable in medicinal chemistry. Key reactions within click chemistry include the Cu-catalyzed azide-alkyne cycloaddition (CuAAC), strain-promoted azide-alkyne cycloaddition (SPAAC), thiol-ene reactions, inverse electron demand Diels–Alder reactions (IEDDA), hydrazone click chemistry, and the recently emerging sulfur fluoride exchange (SuFEx) reaction (Shankaraiah et al., 2019; Kim and Koo, 2019; Barrow et al., 2019). Since its introduction in 2001, click chemistry has become a prominent research focus, driving innovation in the development of bioactive compounds and therapeutic agents (Cai et al., 2023).

#### 2.1.1 Cu-catalyzed azide-alkyne cycloaddition (CuAAC)

**FIGURE 1:**
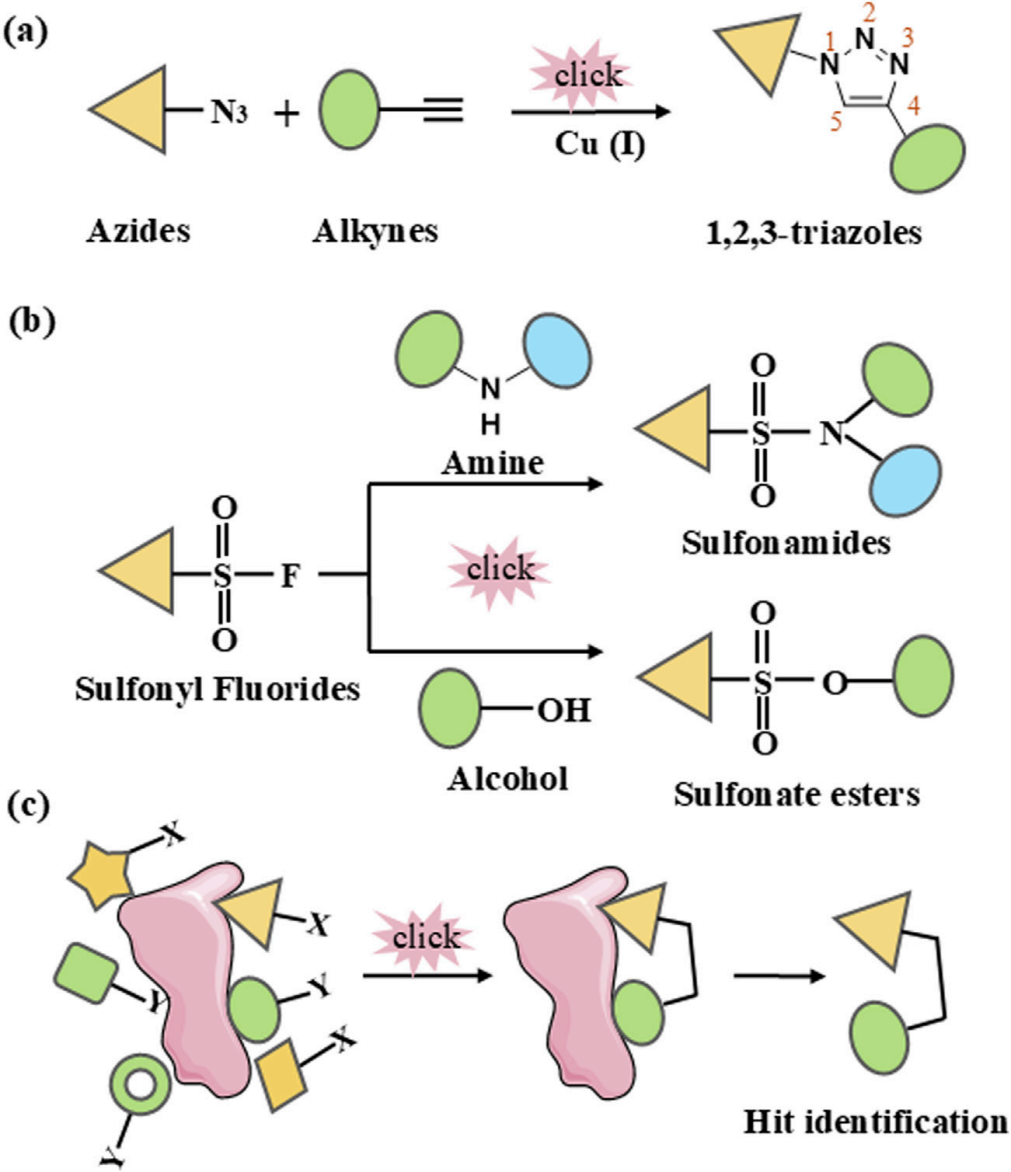
Schematic representation of click chemistry. **(A)** Cu-catalyzed azide-alkyne cycloaddition (CuAAC). **(B)** Sulfur (VI)-Fluoride Exchange (SuFEx). **(C)** *In situ* click chemistry approach.

**FIGURE 2:**
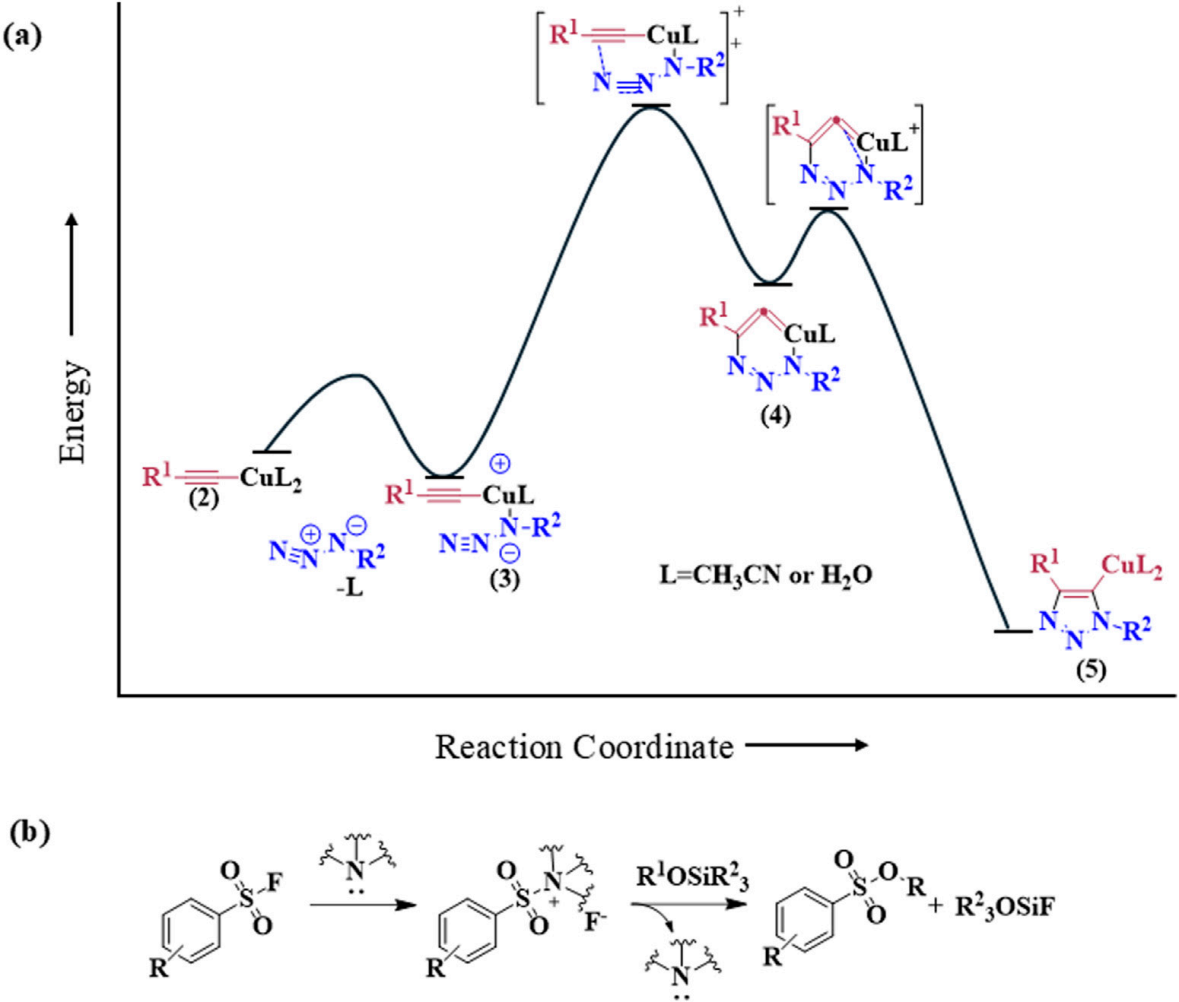
Reaction mechanisms of click chemistry of **(A)** Cu-catalyzed azide-alkyne cycloaddition (CuAAC) and **(B)** Sulfur (VI)-Fluoride Exchange.

**FIGURE 3:**
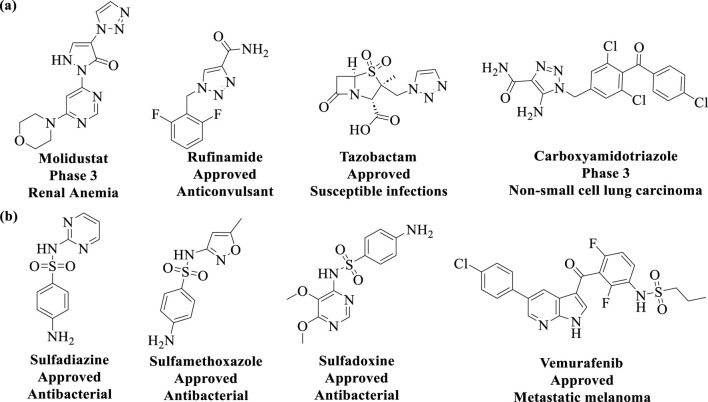
Chemical structures of approved drugs and drug candidates containing the **(A)** 1,2,3-triazole moiety, and **(B)** sulfonamides moiety.

The discovery of Cu(I) catalyzed CuAAC in 2001 represented a pivotal advancement, transforming click chemistry from a theoretical concept into a widely accepted and practical methodology (Kolb et al., 2001). Among all click reactions, CuAAC has emerged as the most popular synthetic and coupling tool. This reaction offers exceptional application potential due to its ability to facilitate the functionalization of organic scaffolds with azides and alkynes (Jiang et al., 2019), which remain stable during subsequent transformations in the presence of highly functionalized biomolecules, molecular oxygen, water, and other common reaction conditions. In contrast to the uncatalyzed cycloaddition of azides and alkynes, which yields a mixture of 1,4- and 1,5-triazole regioisomers at elevated temperatures, CuAAC selectively combines organic azides and terminal alkynes to produce 1,4-disubstituted 1,2,3-triazoles exclusively under mild conditions (Figure 1A), thus eliminating the need to separate the 1,4- and 1,5-linked regioisomers using classical chromatographic techniques. The detailed reaction mechanisms underlying this transformation are illustrated in Figure 2A. Notably, the CuAAC reaction offers key advantages, including exceptional stereoselectivity, rapid reaction rates, and the ability to form intermolecular connections efficiently under mild conditions. These attributes have positioned CuAAC as a powerful and widely adopted tool in drug discovery. Compounds containing the 1,2,3-triazole moiety have demonstrated compelling biological activities, as illustrated by several approved drugs and drug candidates depicted in Figure 3A.

**FIGURE 4:**
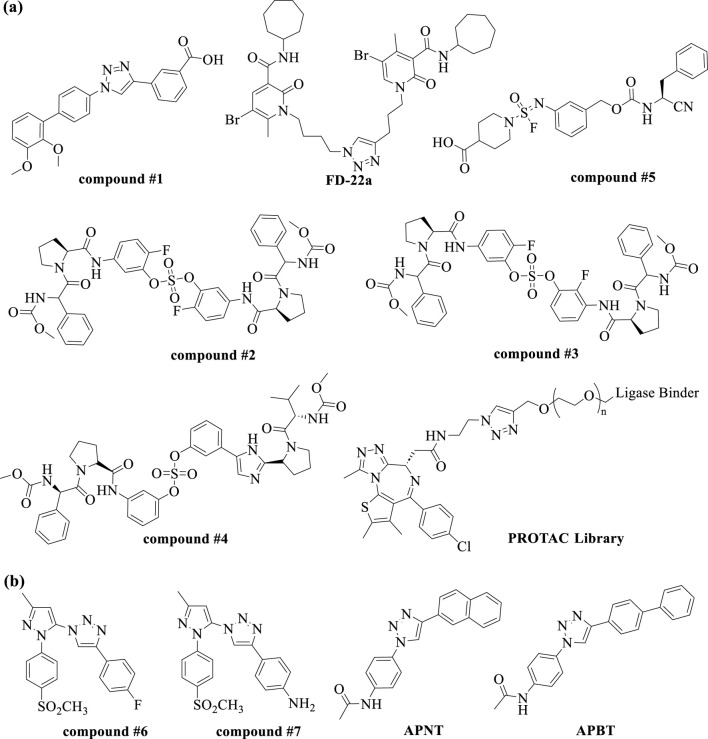
Chemical structures of small molecules discovered from **(A)** the conventional click chemistry and **(B)** *in situ* click chemistry.

Recent studies illustrate the utility of CuAAC click chemistry in drug discovery. For instance, Synta66 has been employed as a chemical probe to elucidate the role of store-operated calcium entry (SOCE) in cellular mechanisms. To identify drug-like SOCE inhibitors with improved pharmacokinetic profiles, Pirali et al. replaced the amide group in Synta66 with a triazole ring. Among the synthesized 1,2,3-triazoles, compound **#1** (Figure 4A) exhibited significantly higher aqueous solubility (1,528 μg/mL vs. 0.28 μg/mL) compared to Synta66. Furthermore, compound **#1** demonstrated potent inhibitory activity at nanomolar concentrations, favorable pharmacokinetic properties, and *in vivo* efficacy in a mouse model of acute pancreatitis (Serafini et al., 2020). In another study, Manera et al. designed, synthesized, and functionally characterized the first bitopic ligands for the cannabinoid receptor CB2 (CB2R). These ligands were synthesized through a click chemistry reaction between azido and alkyne derivatives. The most promising bitopic ligand, **FD-22a** (Figure 4A), exhibited anti-inflammatory activity in a human microglial cell inflammatory model and demonstrated antinociceptive effects *in vivo* in a mouse model of neuropathic pain (Gado et al., 2022). Additionally, Cee et al. reported a click chemistry approach as a reliable linking strategy for the synthesis of a 10-membered library of PROTACs (Figure 4A). This method enables the parallel synthesis of PROTAC libraries, contingent upon the availability of the requisite azides and alkynes. Conceptually, this platform represents a powerful new tool for accessing diverse PROTAC libraries, with principles that can be readily applied to other ligases and target proteins (Wurz et al., 2017).

#### 2.1.2 Sulfur (VI)-fluoride exchange (SuFEx)

In 2014, Sharpless and colleagues introduced the next advancement in click chemistry: SuFEx (Click-II) (Figure 1B). The detailed reaction mechanisms are shown in Figure 2B. SuFEx represents a recent set of ideal click chemistry transformations, characterized by metal-free reaction conditions. This approach utilizes sulfuryl fluoride (SO_2_F_2_) and thionyl tetrafluoride (SOF_4_) to synthesize two key S–F motifs: arylfluorosulfates (Ar–O–SO2–F) and iminosulfur oxydifluorides (R–NSOF2) (Barrow et al., 2019). Although the potential of SuFEx in drug discovery is just beginning to be explored, it holds promise. In contrast to the CuAAC reaction, which has been employed in proof-of-concept studies for lead optimization, only a limited number of drugs feature the 1,2,3-triazole linkage. Sulfonamides, however, are a common structural motif in drug design and represent the primary sulfur-based feature in clinically approved medications (Figure 3B). More than 150 sulfonamide drugs are available on the market (Zhao et al., 2019), encompassing a range of therapeutic activities including antibacterial, antitumor, anti-obesity, anti-thyroid, and analgesic effects for neuropathic pain. Furthermore, sulfonate esters (R–SO_2_–OR) and sulfate diesters (R–OSO_2_–OR) serve as excellent bioisosteric substitutes for carboxylic acids and esters. Despite their greater lipophilicity, these sulfonyl compounds maintain a polarized S–O bond, facilitating robust electrostatic interactions with target proteins (Hong et al., 2019). This positions SuFEx as a promising tool for the modular synthesis of functional libraries in drug discovery.

SuFEx click chemistry has emerged as a valuable tool in drug discovery. Kim, Jang, and colleagues successfully utilized SuFEx reactions in a click chemistry approach to synthesize biaryl sulfate core derivatives, demonstrating their efficacy as potent inhibitors of hepatitis C virus (HCV) nonstructural protein 5A (NS5A). Among the synthesized inhibitors, compounds **#2**, **#3**, and **#4** (Figure 4A) exhibited impressive two-digit picomolar EC50 values against HCV genotype-1b (GT-1b) and single-digit or sub-nanomolar activities against the HCV genotype-2a (GT-2a) strain (You et al., 2018). A pioneering study by Wolan et al. showcased biocompatible SuFEx click chemistry as a proof-of-concept for a high-throughput process aimed at generating drug-like, biologically active molecules. Starting from a modest high-throughput screening hit against the bacterial cysteine protease SpeB, a SuFExable iminosulfur oxydifluoride [RN = S(O)F2] motif was introduced, leading to the rapid diversification into 460 analogs in overnight reactions. Direct screening of these products yielded drug-like inhibitors with up to 300-fold increased potency. Compound **#5** (Figure 4A) is the first potent and selective SpeB inhibitor, useful for studying the protease’s role in cellular and animal models (Kitamura et al., 2020). More recently, the research groups of Moses et al. demonstrated the versatility and practicality of Accelerated SuFEx Click Chemistry (ASCC) for the late-stage derivatization of bioactive molecules. They employed ASCC to synthesize discrete compound libraries in a 96-well plate format, ensuring experimental consistency and significantly enhancing the efficiency of identifying potent hit molecules. Additionally, ASCC was utilized to create arrays of sulfonate ester-linked, high-potency microtubule-targeting agents (MTAs) that exhibited nanomolar anticancer activity against multidrug-resistant cancer cell lines. These findings highlight ASCC’s promise as a robust platform for advancing drug discovery (Homer et al., 2024).

### 2.2 *In situ* click chemistry

The discovery of bioactive compounds hinges on the iterative generation of SAR data derived from the preparation and biological assays of hit congeners. However, traditional methods are often time-consuming and labor-intensive, leading to slow and costly hit-to-lead transitions. Target-guided synthesis (TGS) is a synthetic strategy for enzyme inhibitors that involves the selective assembly of complementary functional groups based on biological targets (e.g., enzymes) serving as templates (Hu and Manetsch, 2010a). The application of click chemistry in kinetic TGS, known as *in situ* click chemistry, represents an innovative synthesis process where a biological target facilitates the assembly of its own inhibitor through the guided selection of suitable building blocks (Hu and Manetsch, 2010a; Bhardwaj et al., 2017) (Figure 1C). This approach not only results in compounds with high binding affinity for the target but also offers a powerful strategy for rapidly preparing and characterizing compound libraries of bioactive molecules, greatly improving the efficiency of drug discovery. Fragment-based drug design plays a crucial role in drug discovery. *In situ* click chemistry provides a unique strategy for fragment modification and optimization. In this approach, chemical ligation depends on the close proximity and optimal spatial arrangement of reactive fragments (Bhardwaj et al., 2017). When these fragments bind simultaneously to the target’s binding sites, they facilitate the formation of irreversible bonds. Consequently, the selection of suitable combinatorial fragment libraries is a critical factor for the success of this method.

The general protocol for assembling and screening focused combinatorial fragment libraries using *in situ* click chemistry involves four key steps. First, suitable fragments are selected, typically featuring a common warhead—a core structure for interaction with a specific class of biological targets. These fragments should exhibit a variety of substituents and functional groups, as greater skeletal and stereochemical diversity within the chemical space increases the likelihood of identifying effective hits. Additionally, the fragments need to possess desirable properties such as stability, solubility, and cellular permeability to ensure compatibility with biological systems (Wang et al., 2016; Rzuczek et al., 2014; Hu and Manetsch, 2010b). Second, the reactant fragments are synthesized to include reactive groups such as azides, alkynes, or sulfonyl fluorides, which serve as warheads in the click chemistry reactions. Third, *in situ* screening is conducted, typically in 96- or 384-well plates, enabling high-throughput screening without further purification, thus streamlining the drug discovery process. Finally, interactions between the fragment molecules and target compounds are assessed based on preliminary screening data, such as percentage inhibition, with promising hits further validated through IC_50_ determination to confirm their potency (Wang et al., 2016).

In recent years, the rapid assembly and *in situ* screening of focused combinatorial fragment libraries using CuAAC and SuFEx click chemistry have emerged as robust and efficient strategies for generating bioactive molecules. In 2015, Wuest and colleagues demonstrated for the first time the use of CuAAC click chemistry in conjunction with the cyclooxygenase-2 (COX-2) active site as a reaction vessel for the *in situ* generation of highly specific inhibitors. This study led to the discovery of two highly potent and selective COX-2 isozyme inhibitors (compounds **#6** and **#7**) (Figure 4B), which exhibited significantly greater *in vivo* anti-inflammatory activity compared to widely used selective COX-2 inhibitors. These findings provide a valuable tool for the economical and rapid screening of potential drug candidates (Bhardwaj et al., 2017). In 2016, Wang and colleagues introduced two cell-permeable O-GlcNAc transferase (OGT) inhibitors, **APNT** and **APBT** (Figure 4B), developed from low-activity precursors (IC50 > 1 mM) through a strategy termed “tethering *in situ* click chemistry” (TISCC). Among these, APNT exhibited a significantly enhanced inhibitory activity, with an IC50 of 66.7 ± 0.8 μM, representing over a 60-fold improvement. Notably, APNT’s potency was comparable to that of benzoxazolinone, an irreversible OGT inhibitor. However, benzoxazolinone’s high reactivity made it unsuitable as a selective OGT inhibitor for cellular applications. In contrast, both **APNT** and **APBT** effectively suppressed O-GlcNAcylation in cells without causing significant cytotoxicity. This highlights TISCC as a promising approach for optimizing low-activity precursors with millimolar IC50 values into potent and selective inhibitors (Wang Y. et al., 2017).

### 2.3 Challenges of click chemistry in drug discovery

Click chemistry offers several advantages, including insensitive to oxygen and water, high yields, regioselectivity, and stereoselectivity. These properties enable researchers to rapidly prepare target compounds and build compound libraries. Furthermore, *in situ* click chemistry leverages enzymes as reaction templates, taking advantage of their favorable conformations under physiologically relevant conditions. This approach selectively connects individual building blocks to synthesize novel enzyme inhibitors. However, the application of click chemistry in drug discovery also presents challenges and should not be regarded as a universal solution. Firstly, when generating large numbers of compounds at a rapid pace, it remains uncertain whether click chemistry consistently yields compounds with desirable drug-like properties. As a result, further optimization of biological activity and drug-like characteristics can also be time-consuming. While copper species are among the simplest and most useful catalysts for the CuAAC click reaction, their introduction into biological systems raises concerns regarding potential toxicity, which may interfere with the screening of compound libraries utilizing biological targets as templates. In contrast, the SuFEx reaction is a metal-free procedure that shows promise for library screening. The synthesis of 1,2,3-triazole rings as pharmacophores or bioisosteres via CuAAC has significant potential in drug design for various diseases. However, the 1,2,3-triazole ring itself is not commonly utilized as a pharmacophore and is rarely found in marketed drugs, indicating certain limitations in its application as a drug molecule. Conversely, sulfonamides are a prevalent feature in drug structures and serve as the primary sulfur-based motif in clinically approved drugs, with over 150 sulfonamide drugs available in the market. Despite their stability, ease of synthesis, and potential as pharmacophores, sulfur-ester linkages remain notably underrepresented in drug molecules. Lastly, despite the decades since its inception, click chemistry encompasses only a limited number of reactions, resulting in the identification of multiple hits and leads for specific targets, but relatively few marketed drugs have been successfully discovered through this approach. Consequently, while click chemistry presents both advantages and disadvantages in medicinal chemistry and drug discovery, it also faces numerous challenges. Ongoing developments hold the promise of advancing this synthetic technique to meet the preclinical and clinical requirements of various systemic and localized diseases.

## 3 Targeted protein degradation (TPD) strategies

Targeting pathogenic proteins with small molecule inhibitors has become a widely adopted strategy for treating various diseases. However, many intracellular proteins are deemed “undruggable” due to the absence of accessible active sites, posing a significant challenge in the design and development of effective small molecule inhibitors (Xie et al., 2023). TPD is an emerging concept in drug discovery, first introduced in 1999 (Wickner et al., 1999). Recently, TPD has gained traction as a novel approach for targeting these previously undruggable proteins, utilizing proteasomal and lysosomal pathways (Ding et al., 2020; Zhang C. et al., 2024). Unlike traditional inhibitors, small molecule degraders do not require continuous exposure of the protein’s binding site, which enhances their therapeutic potential. Additionally, many degraders, such as PROTACs, operate catalytically, allowing for lower dosages to achieve the desired effects (Bondeson et al., 2015). Most TPD strategies, including PROTACs and molecular glues, primarily rely on the ubiquitin–proteasome system (UPS) and predominantly target intracellular proteins. In contrast, lysosome-dependent TPD strategies can degrade membrane proteins, extracellular proteins, and protein aggregates, significantly broadening the range of substrates that can be targeted (Zhao et al., 2022). The proper functioning of these two pathways is crucial for preventing or treating a variety of diseases, including tumors, neurodegenerative disorders, autoimmune diseases, and metabolic syndrome. Therefore, employing proteasomes and lysosomes to target pathogenic proteins represents a promising strategy in addressing these complex health challenges.

### 3.1 Targeted protein degradation via proteasome

The UPS is a crucial pathway for protein degradation, playing a vital role in maintaining cellular homeostasis by facilitating the degradation of over 80% cellular proteins (Chen et al., 2016). This degradation process targets damaged proteins or those that are no longer needed. Ubiquitination is achieved through a cascade involving three key enzymes: an E1 activating enzyme, an E2 conjugating enzyme, and an E3 ligase. The E1 enzyme binds to a ubiquitin molecule in an ATP-dependent manner and subsequently transfers it to the E2 enzyme. The E3 ligase then catalyzes the transfer of the ubiquitin from E2 to the target substrate, leading to the polyubiquitination of the substrate (Eldridge and O'Brien, 2009). The accumulation of the polyubiquitin chain on a targeted protein serves as a signal for degradation by the proteasome. PROTACs and molecular glues are two prominent technologies that leverage the UPS for the targeted degradation of specific proteins of interest (POI), and will be the focus of our discussion.

#### 3.1.1 Proteolysis-targeting chimeras (PROTACs)

**FIGURE 5:**
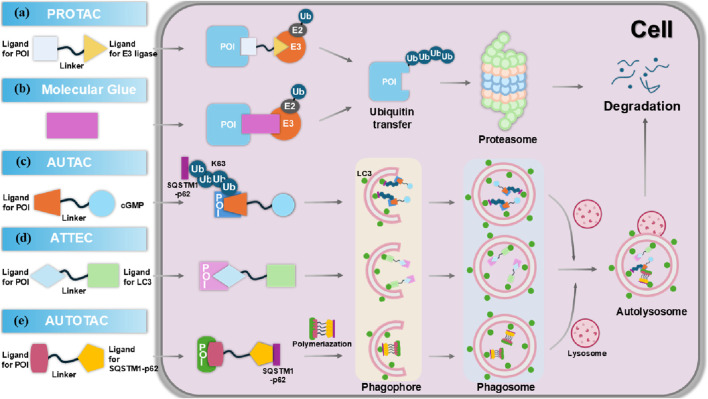
The molecular mechanism of targeted protein degradation (TPD). **(A)** Proteolysis targeting chimeras (PROTACs) are composed of an E3 ligase-targeting ligand, a linker, and a proteins of interest (POI)-binding ligand. By simultaneously interacting with both the POI and the E3 ligase, PROTACs promote the polyubiquitination of the POI, marking it for degradation via the proteasome. **(B)** Molecular glues bind to the E3 ubiquitin ligase, or the POI, facilitating their interaction and triggering the ubiquitination and degradation of the POI. **(C)** Autophagy-targeting chimeras (AUTACs) consist of a POI-targeting warhead, a linker, and a cGMP-based degradation tag. This tag promotes K63 polyubiquitination of the POI, leading to selective degradation via autophagy in the lysosome. **(D)** Autophagosome-tethering compounds (ATTECs) bind LC3 and the POI, **(E)** while AUTOphagy-TArgeting Chimeras (AUTOTACs) bind p62 and the POI, driving autophagosome formation, which then merge with lysosomes to degrade the targeted POI.

**FIGURE 6:**
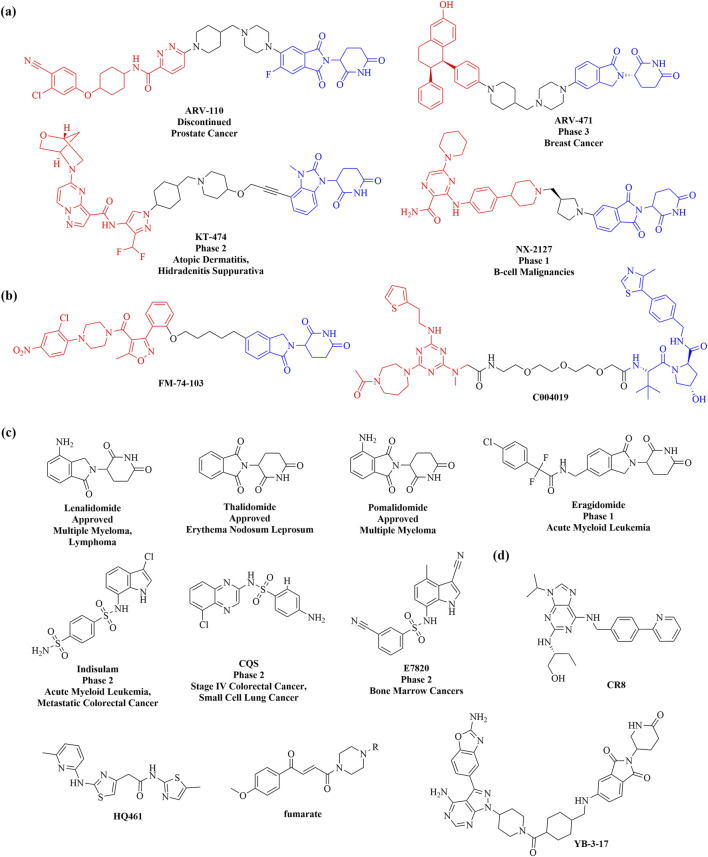
Chemical structures of proteasome-based degraders. Representational PROTACs currently in **(A)** clinical trials and **(B)** preclinical trials. Representational molecular glues currently in **(C)** clinical trials and **(D)** preclinical trials. Degradation tag and proteins of interest (POI) warheads are respectively marked in blue and red.

The UPS, exemplified by PROTACs, offers a powerful strategy for degrading otherwise undruggable POI. PROTACs are synthetic heterobifunctional molecules composed of an E3-recruiting ligand, a POI-targeting warhead, and a flexible linker that connects the two (Konstantinidou et al., 2019). By promoting the formation of a ternary complex comprising the POI, PROTAC, and E3 ligase, these compounds enhance the ubiquitination of the POI, leading to its degradation via the UPS (Figure 5A). Unlike traditional inhibitors, PROTACs operate through an event-driven mechanism, acting as catalysts for selective protein degradation—one molecule of PROTAC can induce the ubiquitination of multiple target protein molecules (Burslem and Crews, 2020). Initially demonstrated by Crews and colleagues in 2001, the concept of PROTACs has since evolved into various innovative degradation technologies targeting kinases, nuclear receptors, epigenetic proteins, misfolded proteins, and even RNAs (Sakamoto et al., 2001; Schreiber, 1992). This broadens the spectrum of potential targets and enhances clinical applications for treating cancer, neurodegenerative diseases, and viral infections. Consequently, PROTAC technology is advancing into clinical settings, with over 15 targeted degraders currently in clinical trials (Figure 6A).

The PROTAC technology represents a promising therapeutic modality for treating various diseases. Here, we highlight some PROTACs that target specific proteins for degradation. A significant achievement in PROTAC technology is the development of orally bioavailable PROTACs that have progressed to clinical trials. Notably, the androgen receptor degrader **ARV-110** (Clinical Trial No. NCT03888612) and the estrogen receptor degrader **ARV-471** (Clinical Trial No. NCT05654623) have entered phase II and III clinical trials for prostate and breast cancer, respectively. The PROTAC approach, widely exploited in cancer research, also holds promise for antiviral development. In 2022, the Marazzi and Jin groups identified **FM-74-103** (Figure 6B), a small-molecule degrader that inhibits infections from Influenza A virus (IAV), syndrome coronavirus 2 (SARS-CoV-2), and cytomegalovirus (CMV). **FM-74-103** selectively degrades human G1 to S phase transition 1 (GSPT1), a translation termination factor, showcasing its potential as a host-directed antiviral that can degrade key factors controlling both RNA and DNA virus replication in host cells. This work highlights the broad utility of the PROTAC platform for rational design and development of next-generation antivirals (Zhao et al., 2023). Moreover, PROTACs can target undruggable proteins, significantly expanding the therapeutic prospects for refractory diseases. Tau protein accumulation is a hallmark of Alzheimer’s disease (AD) and related tauopathies; however, tau is a natively unfolded protein lacking well-defined folds and active sites. In 2021, Wang et al. discovered **C004019** (Figure 6B), a novel small-molecule PROTAC that selectively promotes tau clearance, significantly improving synaptic and cognitive functions in various hTau cell models and in AD-like hTau and 3xTg transgenic mice. This work demonstrates that PROTACs can effectively induce the degradation of undruggable proteins, offering promising therapeutic avenues for AD and related tauopathies (Wang W. et al., 2021).

#### 3.1.2 Molecular glue

The concept of molecular glue was first introduced Harvard chemical biologist Stuart Schreiber in the early 1990s (Schreiber, 1992). As a linker-free scaffold, molecular glues induce, stabilize, or enhance interactions between E3 ligases and their POI by modifying the surface of the E3 ubiquitin ligase, thereby promoting the ubiquitination of the POI (Zhou et al., 2023) (Figure 5B). This modality effectively hijacks the ubiquitin-proteasome pathway to degrade traditionally challenging therapeutic targets, thereby broadening the spectrum of druggable proteins. Unlike PROTACs, molecular glues are generally smaller (typically<500 Da) and lack clearly separable components like linkers or distinct chemical moieties for binding to each protein. While their smaller size and successful precedents suggest that molecular glues may exhibit better drug-like properties, their design poses significant challenges. However, the few molecular glues that have made it into clinical trials were largely discovered serendipitously (Ding et al., 2022) (Figure 6C). In 2014, Krönke et al. reported the first molecular glue, **lenalidomide** (a thalidomide analog), which induces the degradation of IKZF1/3 via the CUL4/CRBN pathway (Krönke et al., 2014). Since the discovery of **thalidomide**, over ten small molecular glues with similar structural motifs have been identified. Subsequent studies revealed that a series of aryl sulfonamides function as molecular glues, enhancing the interaction between the E3 ubiquitin ligase CUL4-DCAF15 (DDB1 CUL4 Associated Factor 15) and RBM39 (RNA binding motif protein 39), thereby promoting RBM39 degradation. Several aryl sulfonamides, including **Indisulam**, **CQS**, and **E7820**, have been evaluated in clinical trials as potential antitumor agents (Figure 6C) (Han et al., 2017; Uehara et al., 2017).

Molecular glue degraders have generated significant interest in the research community. To facilitate the discovery of molecular glue degraders targeting diverse proteins and binding modes, there is an urgent need for new screening methods and rational design strategies that transition from serendipitous discovery to systematic exploration. (1) Innovative Screening Technologies: Ebert et al. developed a method to mine correlations between the cytotoxicity of 4,518 clinical and preclinical small molecules and the expression levels of E3 ligase components across a wide range of human cancer cell lines. This led to the identification of **CR8** as a molecular glue degrader that binds to CDK12–cyclin K, recruiting the DDB1–CUL4–RBX1 E3 ligase complex to ubiquitinate cyclin K (Figure 6D) (Słabicki et al., 2020; Kozicka et al., 2023). Similarly, through chemogenomic screening, Han et al. discovered **HQ461**, a novel molecular glue that binds the ATP pocket of CDK12 and promotes interaction with DDB1, triggering cyclin K degradation (Figure 6D) (Lv et al., 2020). (2) Optimization of Molecular Glue Design: Daniel et al. introduced a transposable chemical handle that can convert protein-targeting ligands into molecular degraders. Using the CDK4/6 inhibitor Ribociclib as a prototype, they identified a covalent handle capable of inducing CDK4 degradation. Further optimization led to the development of a “**fumarate**” handle, a universal modular chemical tool for generating molecular glue degraders (Figure 6D) (Toriki et al., 2023). Additionally, Rao et al. employed a dual-target, dual-mechanism design strategy to combine multiple mTOR inhibitors with E3 ligase ligands. After extensive screening, they discovered the bifunctional molecule **YB-3-17**, which selectively degrades GSPT1 while inhibiting mTOR (Figure 6D) (Liu Y. et al., 2024). These studies demonstrate the growing sophistication of molecular glue degrader design and expand their potential applications across various therapeutic targets.

### 3.2 Targeted protein degradation via lysosome

More recently, new TPD strategies have been developed to hijack the lysosomal degradation pathway, the major degradation pathway independent of the proteasome. As a central “garbage disposal” mechanism within cells, lysosomes facilitate the breakdown of various biomacromolecules, including proteins, lipids, carbohydrates, and nucleic acids, through endocytic, phagocytic, and autophagic pathways (Saftig and Klumperman, 2009). The lysosomal degradation system comprises the endosome/lysosome and autophagy pathways, which operate both independently and synergistically to degrade intracellular and extracellular components. The endosome/lysosome pathway involves a series of membrane-bound compartments that mediate the processing of internalized material. Endocytosed substances are first incorporated into early endosomes, then transported through endosome carrier vesicles, late endosomes, and ultimately delivered to lysosomes for hydrolytic degradation (Ding et al., 2020). Autophagy, an evolutionarily conserved process, plays a critical role in maintaining cellular homeostasis by clearing unnecessary or damaged organelles and proteins via a lysosome-dependent mechanism. In this process, targeted organelles and proteins are encapsulated within double-membrane vesicles, known as autophagosomes (Zhao et al., 2022). These autophagosomes subsequently fuse with lysosomes, where their contents are broken down.

#### 3.2.1 Autophagy-targeting chimeras (AUTACs)

AUTAC is an innovative targeted protein degrader that harnesses the autophagy pathway to facilitate the selective degradation of intracellular proteins and organelle debris (Sakamoto et al., 2001). By inducing specific ubiquitin modifications, particularly K63-linked polyubiquitination, AUTAC molecules create a recognition signal that is effectively processed by the selective autophagy machinery. This K63 polyubiquitination is distinct from the more common K48-linked ubiquitination, which typically signals for proteasomal degradation (Figure 5C) (Takahashi et al., 2019). Structurally, AUTACs are heterobifunctional molecules comprising three key components: a degradation tag, which is often a guanine derivative responsible for initiating ubiquitination; a flexible linker that connects the degradation tag to the ligand; and a ligand warhead that selectively binds to the POI. Following binding, the targeted cellular components are sequestered into autophagosomes, which then fuse with lysosomes for degradation in the presence of lysosomal hydrolases. Despite the promise of AUTAC technology in drug discovery, significant gaps remain in understanding the detailed mechanisms underlying its efficacy, including the molecular interactions involved in the recognition and processing of ubiquitinated targets (Li et al., 2021). Currently, there are no clear examples of AUTACs in clinical trials, with research primarily focused on early preclinical assessments.

**FIGURE 7:**
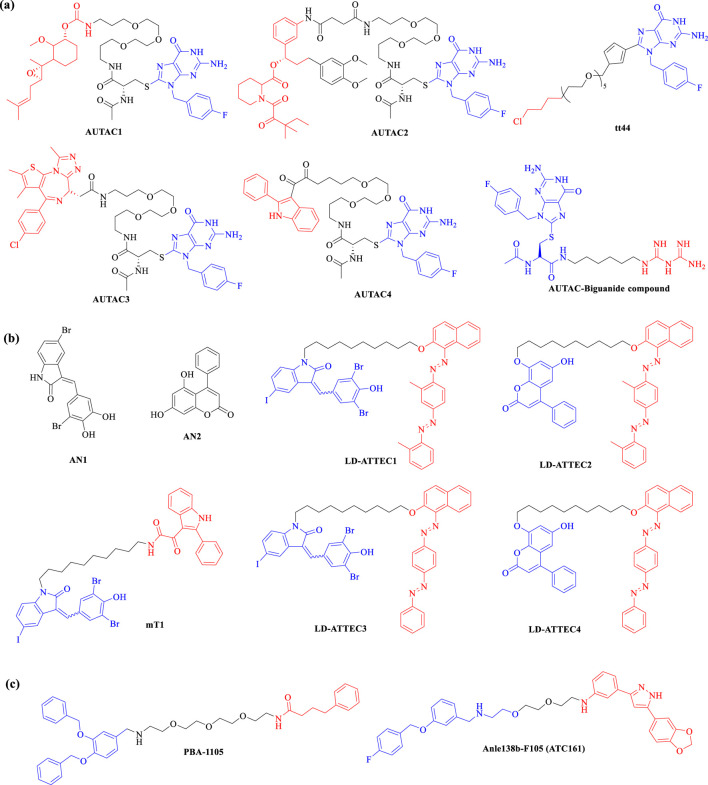
Chemical structures of lysosome-based degraders. Representational **(A)** AUTACs, **(B)** ATTECs and **(C)** AUTOTACs currently in preclinical trials. Degradation tag and proteins of interest (POI) warheads are respectively marked in blue and red.

In 2019, Arimoto’s group introduced the concept of AUTAC, drawing inspiration from the antibacterial autophagy process. Since then, molecules of **AUTACs 1–4** (Figure 7A) have been successfully employed to degrade various proteins, including methionine aminopeptidase 2 (MetAP2), FK506-binding protein (FKBP12), BET family proteins, translocator protein (TSPO), and others, alongside mitochondrial components, showcasing their remarkable degradation capabilities. Subsequent investigations have indicated that the degradation of mitochondria can trigger the elimination of additional pathogenic proteins (Takahashi et al., 2019). In 2023, the group conducted a SAR study, substituting L-Cysteine with pyrazole in the design of second-generation AUTACs. This modification resulted in a substantial enhancement of activity, with the **tt44** (Figure 7A) of second-generation AUTACs achieving up to a 100-fold increase in potency compared to first-generation AUTACs, which were effective at concentrations of 10 μM (Takahashi et al., 2023). More recently, Schmitzer and colleagues developed an **AUTAC-Biguanide compound** (Figure 7A) by incorporating a biguanide functional group as a targeting moiety linked to the guanine scaffold. This strategic addition enables the selective targeting of the degradation process toward mitochondria. Notably, AUTAC-Biguanide exhibits superior antiproliferative properties compared to metformin and demonstrates enhanced selectivity for cancer cells (Vatté et al., 2024). This body of research underscores the potential of AUTAC molecules to effectively degrade cellular organelles, including damaged mitochondria, paving the way for novel therapeutic strategies.

#### 3.2.2 Autophagosome-tethering compounds (ATTECs)

ATTEC was first introduced in 2019 by Professor Boxun Lu of Fudan University (Li et al., 2019). Unlike PROTACs and AUTACs, which rely on ubiquitination for protein degradation, ATTEC molecules operate independently of this modification. Instead, ATTEC compounds function by tethering the POI to LC3, effectively directing disease-related targets to phagocytes for degradation (Figure 5D). This unique mechanism provides broad applicability across various disease contexts and target types (Zhang C. et al., 2024; Li et al., 2019). Distinct from PROTACs and AUTACs, which are characterized by two ligands connected via a linker, ATTEC molecules do not utilize a linker and can be likened to “molecular glue.” This innovative design allows for the precise and efficient recruitment of target proteins. In recent years, a series of novel ATTECs have been developed as heterobifunctional small molecules, enhancing their functionality. Given the diverse range of autophagic substrates, ATTEC technology holds significant promise for extending the applicability of protein degraders to non-proteinaceous biomolecules and organelles. This adaptability may pave the way for new therapeutic strategies targeting a wider array of biological processes, potentially transforming treatment approaches for various diseases.

In 2019, the Lu research group introduced ATTEC technology, developing ATTEC molecules capable of binding both the key autophagy protein LC3 and the mutant huntingtin protein (mHTT). This pioneering study demonstrated that molecules **AN1** and **AN2** (Figure 7B) effectively degrade mHTT in both cellular and *in vivo* animal models, successfully rescuing phenotypes associated with Huntington’s disease (Li et al., 2019). Building on this initial work, Lu and colleagues expanded the application of ATTEC technology by designing small molecules that target lipid droplets (LDs), which are implicated in various metabolic disorders such as obesity, type 2 diabetes, liver steatosis, and atherosclerosis. These novel ATTECs (**LD-ATTECs C1-C4**, shown in Figure 7B) bind to both LC3 and LDs, achieving near-complete clearance of LDs and rescuing LD-related phenotypes in cell cultures as well as in two independent mouse models (Fu et al., 2021). The proof-of-concept findings underscore the potential of ATTECs to facilitate the degradation of LDs. Most recently, Lu’s team has developed new bifunctional ATTEC (**mT1**, shown in Figure 7B) that simultaneously bind to the outer mitochondrial membrane protein TSPO and the autophagosome protein LC3. This innovative approach enhances the engulfment of damaged mitochondria by autophagosomes, promoting their subsequent autophagic degradation (Tan et al., 2023). This study not only confirms the ability of ATTECs to degrade organelles but also presents promising new strategies for intervening in mitochondria-related disorders. Conceptually, this approach could be extended to target a range of other proteins and non-protein entities, broadening the therapeutic potential of ATTEC technology.

#### 3.2.3 AUTOphagy-TArgeting chimeras (AUTOTACs)

In 2022, Kwon’s group introduced AUTOTACs, an innovative degrader technology that degrades UPS-resistant misfolded proteins and their oligomeric/aggregated species (Ji et al., 2022). AUTOTACs directly bind to the ZZ-type zinc finger (ZZ) domain of sequestosome 1 (SQSTM1)/p62, eliminating the need for polyubiquitination. This bifunctional molecule comprises two key components: a ligand that targets the POI and a ligand that directs the molecule to the autophagy pathway via the p62 protein (Figure 5E). By effectively linking these components through a flexible linker, AUTOTACs facilitate the simultaneous degradation of the target protein while enhancing autophagic flux. This dual functionality not only improves the efficiency of protein degradation but also promotes cellular health by ensuring the clearance of damaged or unnecessary proteins through autophagy, offering a promising strategy for therapeutic interventions in various diseases.

In 2022, the research team led by Yong Tae Kwon developed AUTOTAC compounds that features a module specifically designed to interact with the ZZ domain of p62, alongside a module targeting the POI. Both *in vitro* and *in vivo* studies demonstrated that these AUTOTACs (e.g., **PBA-1105**, **Anle138b-F105**, shown in Figure 7C) effectively targets the androgen receptor (AR) in prostate cancer cells and methionine aminopeptidase 2 (MetAP2) in glioblastoma cells, showcasing its powerful degradation capabilities (Ji et al., 2022). Building on this success, in 2023, Kwon’s group synthesized a series of AUTOTAC molecules that bind both p62/SQSTM1 and α-synuclein aggregates, which are implicated in the progression of Parkinson’s disease. One notable compound, **ATC161** (Figure 7C), is an oral drug characterized by favorable pharmacokinetic and pharmacodynamic profiles. It selectively induces the degradation of α-synuclein aggregates in both *in vitro* and *in vivo* models (Lee et al., 2023). This research underscores the potential of the AUTOTAC platform for advancing drug discovery in the context of proteinopathies and related diseases.

### 3.3 Challenges of targeted protein degradation in drug discovery

TPD has emerged as a groundbreaking modality in drug discovery over the past 2 decades, with PROTACs and molecular glues representing the most advanced technologies in this field. In recent years, lysosome-based degrader technologies have begun to gain traction, significantly expanding the range of disease-related targets that can be addressed, thereby offering new strategies for clinical treatment. However, the development of lysosome-based TPD technologies remains in its infancy compared to the more established PROTAC and molecular glue approaches. While multiple PROTAC compounds have shown great promise in clinical trials, their development is not without challenges. First, synthesizing PROTACs requires not only the identification of a suitable ligand for the protein of interest but also converting that ligand into a functional PROTAC, which can be a time-intensive process demanding considerable expertise in synthetic and medicinal chemistry. Additionally, PROTACs often struggle with issues related to cell permeability and oral bioavailability, primarily due to their complex chemical structures. Furthermore, traditional methodologies do not provide a comprehensive understanding of the mechanisms of action of PROTACs, necessitating the implementation of complementary technologies for thorough evaluation. In contrast, molecular glues are generally smaller and more drug-like, which enhances their potential for clinical application. However, significant challenges remain, particularly during the later stages of optimization. While lysosome-based TPD technologies are still emerging, further research is essential to elucidate the molecular mechanisms and SAR of AUTACs, ATTECs, and AUTOTACs, and to establish their fundamental design principles. Despite these hurdles, TPD technologies hold immense potential to not only serve as powerful tools for biomedical research but also to enrich the medicinal chemist’s toolkit. With focused research and development efforts, these technologies could substantially broaden the spectrum of degradable targets, paving the way for exciting new avenues in therapeutic discovery.

## 4 DNA-encoded libraries (DELs) technology

**FIGURE 8:**
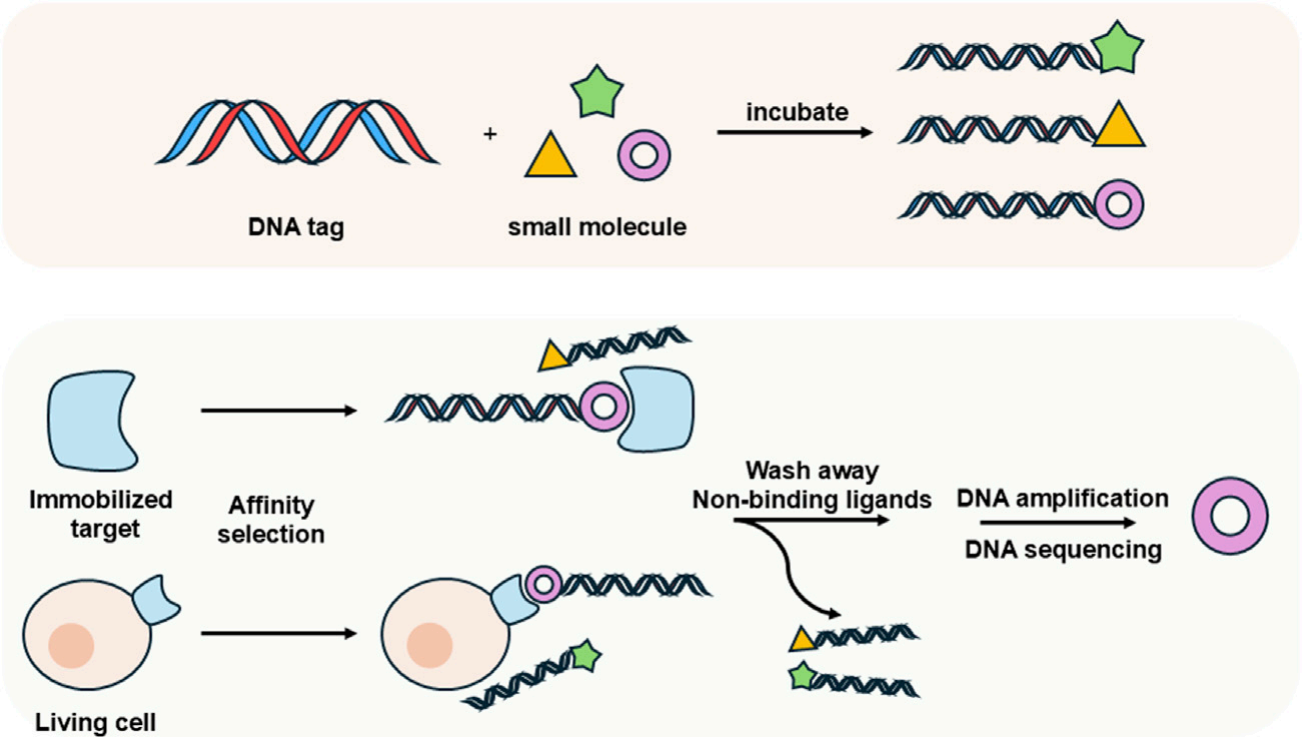
Schematic illustration of DNA-encoded libraries (DELs).

DELs have emerged as a revolutionary tool in drug discovery, offering significant advantages in the identification of potential therapeutic candidates. Initially conceptualized by Brenner and Lerner in 1992, who proposed a theoretical framework for utilizing DNA to encode synthetic peptides (Brenner and Lerner, 1992), DEL technology saw practical application when Liu and Gartner employed DNA-templated synthesis to create small molecules with diverse chemical structures in 2001 (Gartner and Liu, 2001). DELs consist of combinatorial libraries of drug-like molecules, each uniquely barcoded with a DNA sequence that encodes the structure of the attached library member (Clark et al., 2009; Potowski et al., 2021). This innovative approach enables the preparation of libraries with unprecedented diversity and facilitates efficient screening for ligands that bind to specific protein targets. Traditional methods of synthesizing and screening individual molecules are often costly and complex, requiring substantial quantities of target protein, well-established bioassays, and intricate logistical arrangements. In contrast, DEL selections can be performed in just a few days, utilizing only tens of micrograms of protein and picomole amounts of DELs, along with next-generation DNA sequencing (NGS) technologies (Matsuo et al., 2023) (Figure 8). Compared to conventional compound libraries, DELs offer higher density, improved stability, and reduced costs, thereby opening up new avenues for the design and synthesis of complex molecules. This capability not only enhances the efficiency of the drug discovery process but also expands the range of chemical diversity that can be explored, ultimately contributing to the development of more effective therapeutics.

### 4.1 Selections on single immobilized targets

The application of DELs for small molecule discovery primarily involves affinity-based selections conducted on single immobilized targets *in vitro*. In these affinity-based selections, purified proteins equipped with affinity tags are immobilized on solid matrixes, such as magnetic beads or resin (Cochrane et al., 2021; Zhang Y. et al., 2024). This setup enables the effective separation of active library members that bind to the target from those that are weakly or non-binding and remain in solution. Following this, a series of washing steps is employed to remove inactive library members, ensuring that only those bound to the target remain attached. The target-bound library members can be eluted through various methods, including cleaving the target from the solid support, disrupting ligand-target interactions, or introducing an excess of a competitive ligand. In the context of DELs, the DNA sequences encoding the selected library members are then amplified via PCR. This amplified DNA can be subjected to additional rounds of *in vitro* selection, often under increasingly stringent conditions, to enrich the most active compounds (Scheuermann et al., 2006; Neri and Lerner, 2018). This straightforward yet effective methodology allows for the rapid evaluation of molecules with affinity for a target of interest. As such, it serves as a crucial starting point for further optimization, characterization, and eventual development of candidates for clinical trials. By leveraging the unique advantages of DEL technology, researchers can streamline the drug discovery process and enhance the likelihood of identifying promising therapeutic candidates.

**FIGURE 9:**
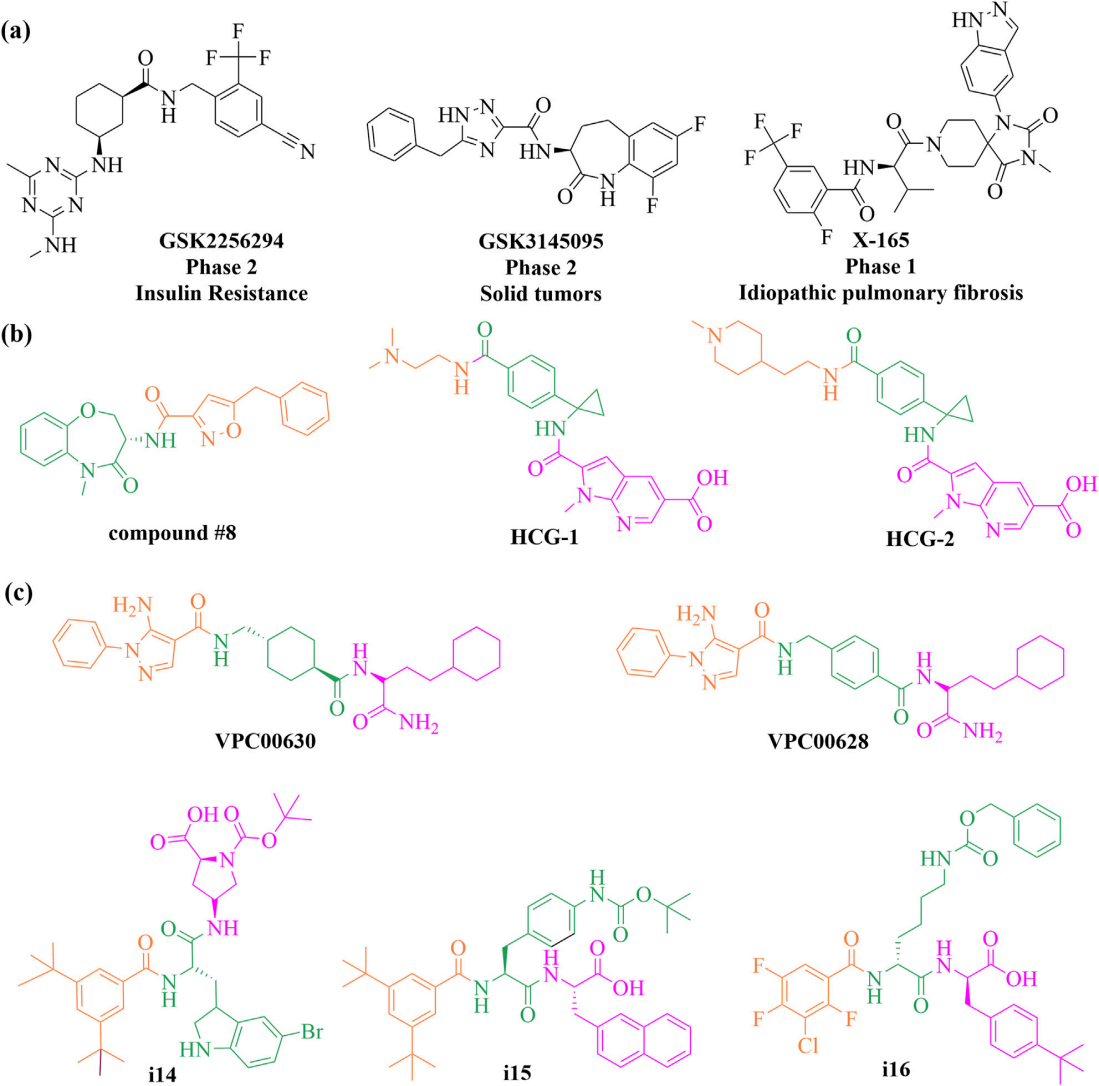
Chemical structures of small molecules discovered from DNA-encoded libraries (DELs). Representational small molecules currently in **(A)** clinical trials, and **(B, C)** preclinical trials.

Traditional workflows for DELs have proven effective in identifying multiple clinical candidates, with more than three candidates having progressed to clinical trials to date (Figure 9A). Gough, Bertin, and their collaborators developed extensive DNA-encoded small-molecule libraries using a split-and-pool strategy, resulting in approximately 7.7 billion diverse warheads across three cycles of building blocks. Utilizing these libraries, they discovered a novel series of highly potent benzoxazepinone inhibitors targeting receptor interacting protein 1 (RIP1) kinase. Notably, these inhibitors (e.g., compound **#8**, shown in Figure 9B) demonstrated complete selectivity against over 450 off-target kinases in a stereochemical-dependent manner. This series not only showcases high potency and kinase selectivity but also exhibits favorable pharmacokinetic profiles in rodent models (Harris et al., 2016). Building on this success, the team optimized a benzoxazepinone hit from their DNA-encoded library, leading to the discovery of the clinical candidate GSK2982772, which is currently undergoing phase 2 clinical trials for psoriasis, rheumatoid arthritis, and ulcerative colitis (Harris et al., 2017; Weisel et al., 2020). In a significant expansion of DEL technology, Dou et al. in 2022 adapted the approach for RNA targets. They initially faced challenges with false positive signals arising from unintended DNA-RNA interactions during their selection against the HIV-1 TAR (trans-acting responsive region) RNA. To mitigate this issue, they developed an optimized strategy incorporating RNA patches and competitive elution to minimize unwanted binding. Subsequent k-mer analysis and motif searches effectively filtered out false positives. This refined method was successfully applied to a DEL selection against the *Escherichia coli* FMN riboswitch, yielding compounds (e.g., **HGC-1** and **HGC-2**, shown in Figure 9B) with nanomolar binding affinities and comparable potency in functional assays (Chen et al., 2022).

### 4.2 Selections on living cells

Traditional DELs selection methods that rely on purified, immobilized protein targets face significant limitations when applied to many critical classes of drug targets. Certain proteins cannot be isolated or subjected to *in vitro* selection due to instability or the absence of established isolation protocols. Additionally, conventional DELs screening is conducted in dilute solutions containing primarily water, buffers, and salts, which fails to accurately replicate the complex cellular environment. The cytosol of living cells comprises 20%–30% (by weight) diverse macromolecules, creating a markedly different milieu compared to the dilute conditions used in traditional screening (Ellis, 2001). Consequently, there is a pressing need to perform ligand discovery directly in live cells (Figure 8). Developing approaches that enable the selection of DELs against targets within a cellular context would eliminate the challenges associated with requiring a pure, active protein and allow for the assessment of proteins in a more functionally relevant state. This would ensure that essential binding partners and post-translational modifications are preserved, ultimately leading to more effective drug discovery outcomes.

The Hansen group achieved a significant milestone by successfully screening a multimillion-member DELs within living cells, utilizing oocytes from the South African clawed frog, *Xenopus laevis*. They screened a DEL comprising 194 million members against three protein targets: mitogen-activated protein kinase 14 (p38α), human acetyl-coenzyme A synthetase 2 (ACSS2), and human dedicator of cytokinesis 5 (DOCK5). This effort yielded multiple chemical clusters and identified nanomolar-level potent hits (e.g., **VCP00630** and **VCP00628**, shown in Figure 9C) for p38α, highlighting the potential of DELs to broaden target screening and reduce attrition rates in drug discovery (Petersen et al., 2021). In a separate study, a novel approach was introduced for identifying small molecule agonists for membrane proteins by selecting DELs on live cells. This method connects extracellular ligand binding to intracellular biochemical responses, thereby enhancing the likelihood of discovering agonists. It was demonstrated on three membrane proteins: epidermal growth factor receptor (EGFR), thrombopoietin receptor (TPOR), and insulin receptor (INSR), utilizing DELs containing 30 million and 1.033 billion compounds. This innovative approach successfully identified novel agonists (e.g., **i14**, **i15** and **i16**, shown in Figure 9C) with sub-nanomolar affinities and micromolar cellular activities (Huang et al., 2024).

### 4.3 Challenges of DNA-encoded libraries in drug discovery

DELs present significant advantages in drug discovery, but they also face notable challenges. One major challenge is the delivery of DELs into target cells, as traditional methods often struggle with membrane permeability, limiting the effective screening of compounds *in vivo*. While advances have been made using techniques like electroporation and microinjection, achieving efficient delivery while maintaining cell viability remains a hurdle. Another issue is the complexity of interpreting the binding interactions between DEL members and target proteins. High background noise from endogenous proteins can confound results, making it difficult to distinguish between specific and nonspecific interactions. Additionally, the large size of DELs complicates the characterization of individual hits, as the multimeric nature of the libraries can obscure individual ligand properties. Furthermore, ensuring the stability and integrity of the DNA during the selection process is crucial, as degradation can affect the quality of the results. Lastly, while DELs enable high-throughput screening, the need for extensive bioinformatics resources to analyze and validate hits can be resource-intensive, posing a logistical challenge for many research teams. Addressing these challenges is essential for maximizing the potential of DELs in drug discovery and expanding their applicability to a broader range of targets.

## 5 Computer-aided drug design (CADD)

**FIGURE 10:**
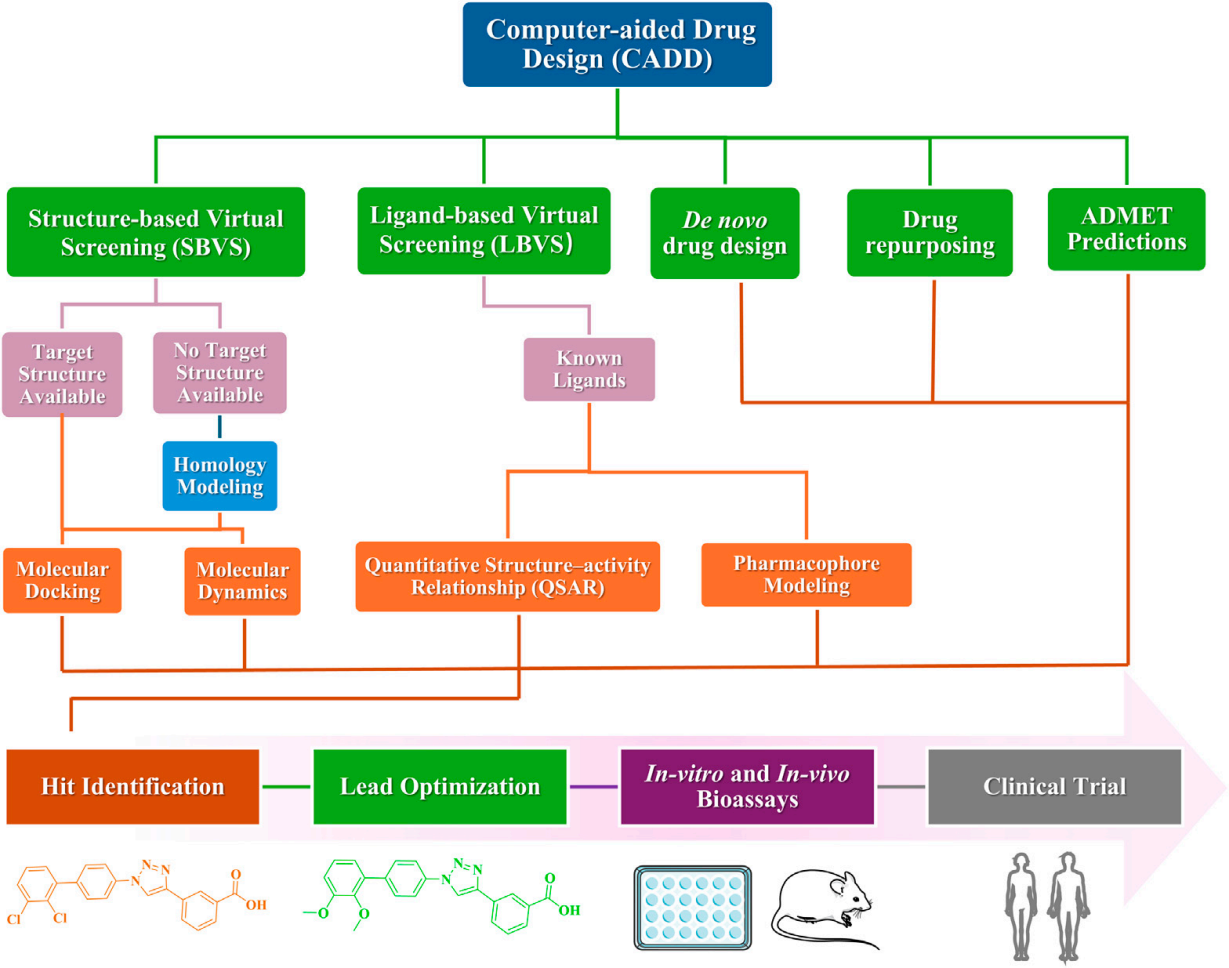
A typical workflow for computer-aided drug design (CADD).

**FIGURE 11:**
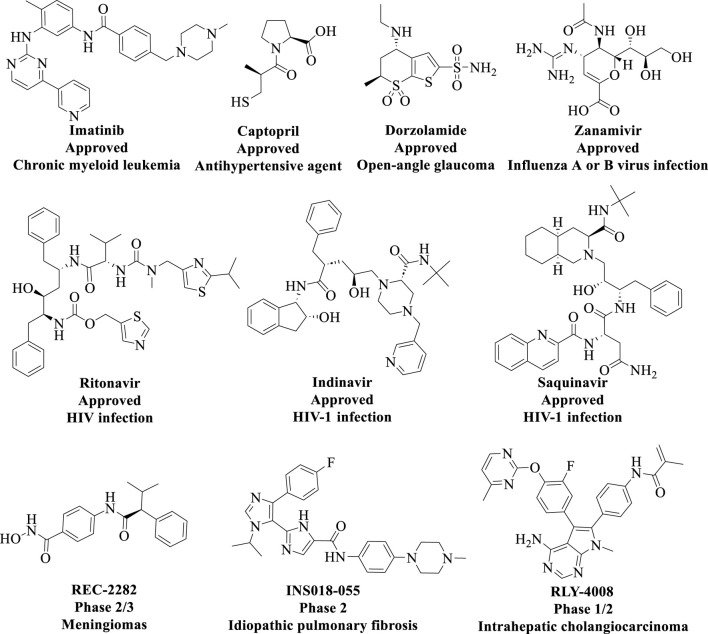
Chemical structures of approved small molecules discovered from CADD technology.

In recent years, significant advancements have been made in the pharmaceutical field, driven by the rapid development of computer technology and artificial intelligence (AI). The policy for the evaluation of chemicals (Registration, Evaluation and Authorisation of Chemicals [REACH]) has strongly advocated for the adoption of *in silico* methods within the pharmaceutical industry (Neri and Lerner, 2018). This approach is particularly justified, as it enables a substantial reduction in the use of laboratory animals for *in vivo* testing and leads to considerable cost savings in research. Recent studies have established CADD is an indispensable tool for drug discovery and can speed up, especially, the early development stages of lead identification and optimization (Rusinko et al., 2024; Hsieh et al., 2023) (Figure 10). CADD primarily integrates traditional disciplines such as computational chemistry, molecular biology, and structural biology. By utilizing advanced algorithms and predictive models, it facilitates the assessment of interactions between drug molecules and biomolecules, thereby accelerating the drug design and optimization processes. Consequently, several small molecules discovered through CADD technology have been approved, as illustrated in Figure 11. Additionally, AI technologies—including machine learning (ML), deep learning (DL), and natural language processing—have been successfully implemented in real-world drug discovery (Liu G. et al., 2023; McNair, 2023).

### 5.1 Structure-based virtual screening (SBVS)

In structure-based drug design (SBDD), the binding interaction and binding site between target structure and ligand are determined using various techniques such as X-ray diffraction, NMR, or calculations involving molecular mechanics and dynamics techniques (Zhu et al., 2020; Sugiki et al., 2018; Defelipe et al., 2018). However, in SBVS, basing a three-dimensional (3D) structural model of the intended target macromolecule (usually a protein, or RNA structure) search and rank accessible chemical space for potential ligands (Maia et al., 2020). SBVS is widely employed to find small molecules that can bind to the active or allosteric site of the target, predicting potential drug candidates. SBVS involves docking of large and diverse virtual chemical libraries into an X-ray crystal structure or homology model of the target protein. The success of SBVS depends on the accuracy of both the target structure and the docking/scoring algorithms (Macalino et al., 2015). The integration of AI technologies in SBVS has influenced the field by improving the accuracy, speed, and scalability of virtual screening (Lima et al., 2016; Zhang et al., 2023).

#### 5.1.1 Molecular docking

The most employed SBVS technique in drug design studies is molecular docking. This method is a computational technique used to predict how small molecules (ligands) interact with a target macromolecule, typically a protein. Nowadays more than 227,000 biological macromolecular structures are deposited on Protein Data Bank (PDB) database (http://rcsb.org), covering more than 170,000 organisms and 190,000 distinct protein sequences. Molecular docking techniques have been popular in SBVS since the early 1980s (Kuntz et al., 1982). Molecular docking study prophesies the interaction energy between two molecules, and determines the interactions of ligand and target to find the conformation of ligand in the formed complex with overall binding free energy (Forli et al., 2016). In general, the process of molecular docking is performed according to the following steps: (i) obtain the 3D structure of the target macromolecule from crystal structure repositories (e.g., PDB); (ii) characterization of the binding sites and cavities; (iii) compound library construction, which involves several processes of characterization, filtering, and clustering; (iv) molecular docking of target with compounds supplemented with known actives and decoys followed by scoring; and (v) final evaluation and validation (Lionta et al., 2014). Some common tools are used in molecular docking, such as AutoDock and AutoDock Vina, Glide, GOLD (Genetic Optimization for Ligand Docking), DOCK, Surflex-Dock, LeDock and FlexX (Cheng et al., 2012; Shen et al., 2020). Moreover, AI-based enhancements in scoring, protein flexibility modeling, and ligand design have transformed molecular docking into a more efficient and reliable tool for drug discovery, making it possible to screen vast chemical libraries and optimize lead compounds with high precision. Molecular docking are superseded by tools like AtomNet, DeltaVina (AI-enhanced AutoDock Vina) and GNINA (Stafford et al., 2022; Wang and Zhang, 2017; McNutt et al., 2021).

**FIGURE 12:**
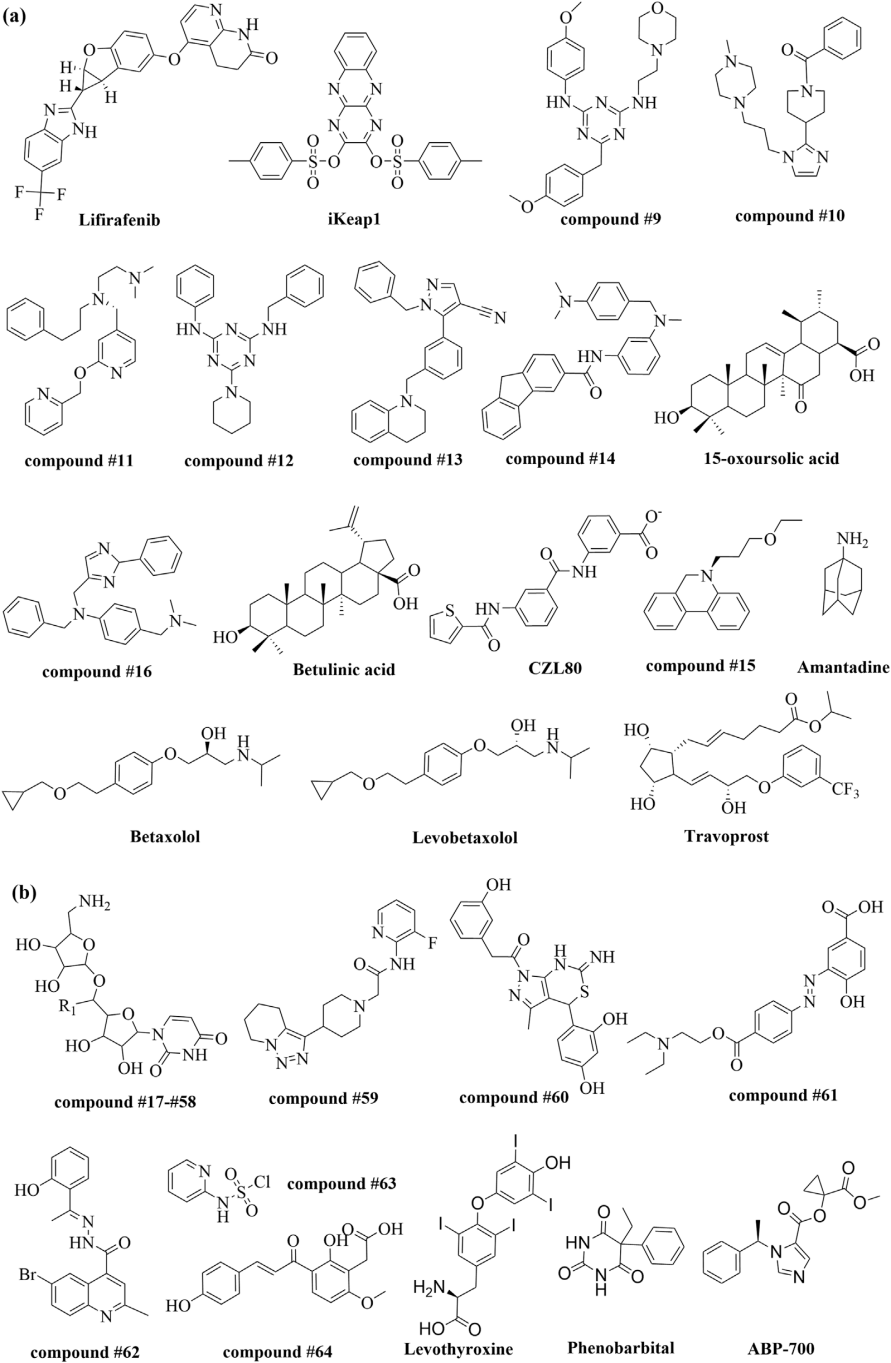
Chemical structures of small molecules discovered from virtual screening. **(A)** Exemplary small molecules by structure-based virtual screening (SBVS). **(B)** Exemplary small molecules by ligand-based virtual screening (LBVS).

In 2019, Xie et al. conducted a virtual screening of 2,004 clinical and preclinical drugs using AutoDock Vina to target multiple enzymes. Their study identified kinase inhibitors, particularly **Lifirafenib** (Figure 12A), as promising lead compounds for inhibiting the aroQ, phzG, and phzS enzymes, which are involved in the phenazine biosynthesis pathway. These findings highlight the potential of **Lifirafenib** in drug repurposing, though further experimental validation is necessary (Xie and Xie, 2019). The combination of molecular docking and drug repositioning strategies is proving effective in accelerating the discovery of new preclinical drug candidates. SBVS has been shown to enhance hit quality, especially as the number of compounds screened increases. Arthanari et al. developed VirtualFlow, an open-source platform for large-scale virtual screening capable of handling ultra-large libraries and multiple docking programs. VirtualFlow processed over one billion compounds using AutoDockTools, leading to the discovery of lead molecules such as **iKeap1** (Figure 12A), which binds to Kelch-like ECH-associated protein 1 (KEAP1) with nanomolar affinity, demonstrating significant potential in drug discovery (Gorgulla et al., 2020). In 2023, Houston et al. introduced SCORCH, a new ML-based model designed to improve the scoring functions used in molecular docking. SCORCH showed superior performance in virtual screening and pose prediction compared to traditional scoring methods, with higher enrichment factors and improved pose ranking. This model’s increased accuracy, combined with uncertainty estimation, offers a promising approach to reducing drug discovery costs and timelines (McGibbon et al., 2023).

#### 5.1.2 Homology modeling

An interesting contradiction in drug discovery is the fact that 60%–70% of drugs on the market target integral membrane proteins, which to date have been unsuitable for structure-based approaches because of the enormous difficulties in producing suitable quantities of functional protein for crystallization trials (van Montfort and Workman, 2009). In the case of target proteins for which no direct crystallographic data are available, the construction and screening of homology models can be performed. Homology modeling is a computational method used to predict the 3D structure of a protein based on its amino acid sequence and an available structure of a homologous protein (a protein with a similar sequence). The homology modeling consists of five key steps: (i) find a suitable homologous protein structure to use as a template, (ii) align the sequence of the target protein with the sequence of the template protein, (iii) construct a 3D model of the target protein based on the template structure, (iv) improve the quality of the modeled structure, and (v) assess the quality and accuracy of the model (Lee et al., 2017). Homology modeling employs information from template structures of homologous proteins. Some common tools include BLAST, MODELLER, SWISS-MODEL, and PyMod (Bullock et al., 2013; Waterhouse et al., 2018; Janson and Paiardini, 2021). However, with the advance in the AI field, these methods are superseded by methods like AlphaFold, RoseTTAFold, ESMFold, and OmegaFold, which supersedes traditional homology modeling methods by offering greater accuracy, faster predictions, and the ability to model proteins for which no template is available (Lee et al., 2022; Song et al., 2024; Chen et al., 2024).

Overexpression of Forkhead box protein C2 (FOXC2) is associated with cancer progression and metastasis, making it a key target for anticancer drug development. In 2023, Tao et al. used the I-TASSER server for homology modeling to construct a 3D structure of full-length FOXC2. Ligand-based drug design (LBDD) identified MC-1-F2 analogues from 15 million compounds in the ChEMBL and ZINC databases, resulting in eight promising lead compounds (compounds **#9-#16**) (Figure 12A) through molecular dynamics (MD) simulations and molecular mechanics/generalized Born surface area (MM/GBSA) MM/GBSA calculations (Ibrahim et al., 2022). In 2024, Ali et al. utilized SWISS-MODEL to generate a homology model of the dengue virus non-structural 4B (NS4B) protein and validated it using multiple tools. Molecular docking revealed significant binding affinities of triterpenoids, including **15-oxoursolic acid** and **betulinic acid** (Figure 12A), against NS4B (Ali et al., 2024). The same year, Darvishi et al. employed MODELLER 9v10 and AutoDock Vina for homology modeling and molecular docking to optimize Yarrowia lipolytica L-asparaginase. Three mutants—T171S, T171S-N60A, and T171A-T223A—exhibited increased affinity for L-asparagine and reduced affinity for L-glutamine. This study underscores the utility of combining homology modeling and molecular docking to identify therapeutic candidates with fewer side effects, providing valuable insights for enzyme optimization in drug development (Darvishi et al., 2024).

With advancements in AI, traditional homology modeling methods are being increasingly superseded by ML approaches. Svenningsson et al. compared virtual screenings using protein structures generated by AlphaFold, a ML method, and traditional homology modeling. By docking over 16 million compounds onto the trace amine-associated receptor 1 (TAAR1) model, they selected 30 and 32 highly ranked compounds from the AlphaFold and homology model screenings, respectively. The hit rate of AlphaFold screening (60%) was more than twice as high as that of the homology model, leading to the discovery of the most effective agonist (Díaz-Holguín et al., 2024). RoseTTAFold, inspired by AlphaFold, was tested by Tseng et al. on a collection of solved G-protein-coupled receptor (GPCR) structures in the PDB. They assessed the accuracy of the predictions using the root-mean-square deviation (RMSD) of backbone alpha-carbons, finding that AlphaFold outperformed RoseTTAFold in top-scored model accuracy, while RoseTTAFold showed a smaller variance in RMSDs (Lee et al., 2022). Meanwhile, single-sequence-based structure prediction methods, such as ESMFold and OmegaFold, offer a balance between inference speed and prediction accuracy. Lin et al. introduced ESMFold, a pre-trained language model for fast and accurate structure prediction, achieving accuracy comparable to AlphaFold2 while reducing inference time by up to 60 times (Lin et al., 2023).

#### 5.1.3 Molecular dynamics (MD) simulations

MD simulations are a crucial computational tool either before or after docking to study deep insights into the dynamic interactions between potential drug molecules (ligands) and their target proteins (Liu et al., 2018). MD simulations have been long proposed to provide insight into protein dynamics beyond that available crystallographically, and unravel novel cryptic binding sites, expanding the druggability of the targets (Kuzmanic et al., 2020). MD simulations study protein-ligand interactions, facilitating SBDD and optimizing lead compounds by assessing binding affinities and stability. The MD process involves several key steps: (i) preparing the system (selecting molecules, solvation, and ionization), energy minimization, equilibration under controlled conditions, and conducting a production run where atomic positions are updated over time, (ii) data is collected for analysis, focusing on structural changes, binding interactions, and dynamic properties, and (iii) visualization tools are used to interpret results, leading to insights into molecular behavior and interactions in drug discovery (Yelshanskaya et al., 2020; Tang Y. et al., 2020). MD simulations utilize a variety of tools and software packages, with common examples including GROMACS, CHAPERON *g*, NAMD, VMD, and OpenMM (Yelshanskaya et al., 2020; Yekeen et al., 2023; Phillips et al., 2020; Eastman et al., 2017).

**Amantadine** (Figure 12A) has emerged as a promising candidate for COVID-19 treatment by inhibiting the ion channel activity of SARS-CoV-2 Proteins E and open reading frame (ORF) Protein 10. Rosenkilde et al. demonstrated that **amantadine** blocks Protein E-mediated currents, further elucidating its specific interaction profile through solution NMR and MD simulations (Toft-Bertelsen et al., 2021). Chen et al. identified caspase-1 as a key regulator in febrile seizures (FS), with caspase-1 knockout mice showing resistance to FS. Overexpression increased susceptibility, and MD simulations coupled with MM/GBSA binding free energy calculations identified **CZL80** (Figure 12A) as a potent brain-penetrable, low molecular weight caspase-1 inhibitor. These findings emphasize the utility of MD simulations in virtual screening to uncover drug mechanisms (Tang Y. et al., 2020). Another study combined MD simulations and patch clamp electrophysiology to assess drug candidates targeting K_IR_6.2 wild-type (WT) and developmental delay, epilepsy, and neonatal diabetes (DEND) syndrome-related mutant channels. **Betaxolol** and **Levobetaxolol** (Figure 12A) were identified as effective pore blockers, exhibiting IC50 values ranging from 22 to 55 μM. **Travoprost** (Figure 12A) demonstrated the strongest inhibitory effect on WT and L164P channels. The MD simulations provided insight into the potential channel interaction and clarified the possible mechanisms shed light on mechanisms of the tested drug candidates (Houtman et al., 2022).

### 5.2 Ligand-based virtual screening (LBVS)

LBVS is a valuable computational approach in drug discovery, particularly useful when structural information on the target protein is unavailable (Yang et al., 2019). The core principle of LBVS involves analyzing the binding affinities and structural features of known ligands to design drug molecules with improved affinity and selectivity. Key methodologies in LBVS include QSAR modeling, which links chemical structures with biological activity, and pharmacophore modeling, which identifies critical spatial features necessary for binding (Lee et al., 2011). LBVS approach is especially beneficial in the early stages of drug discovery, enabling efficient screening of large compound libraries and prioritizing candidates for experimental testing. However, the effectiveness of LBVS depends on the availability of high-quality ligand data and may overlook potential drug candidates that lack significant structural similarity to known ligands (Pérez-Castillo et al., 2014). The integration of AI technologies has greatly enhanced LBVS by improving its accuracy and efficiency. ML and DL techniques are used to analyze large datasets, predict molecular interactions, and optimize ligand properties, allowing for more efficient exploration of chemical space and accelerating the identification of promising therapeutic agents (Bonanno and Ebejer, 2019; Wu et al., 2024).

#### 5.2.1 Quantitative structure–activity relationship (QSAR)

QSAR models predict biological activity—such as toxicity, efficacy, or binding affinity—based on molecular properties like electronic, topological, steric, and hydrophobic features derived from a compound’s structure (Vyas et al., 2021). These models aid in designing compounds with improved therapeutic profiles. QSAR uses statistical methods such as multiple linear regression, support vector machines, and neural networks to correlate molecular features with biological outcomes (Tang W. et al., 2020). The QSAR modeling process involves several steps: (i) collecting compound data, (ii) representing molecular structures via descriptors, (iii) preprocessing the data, building and training the model using statistical techniques, and (iv) validating the model through internal and external validation methods (Lee et al., 2011). QSAR modeling employs tools like CoMFA, CoMSIA, HQSAR, Dragon, and PaDEL-Descriptor for molecular descriptor calculation (Sainy and Sharma, 2015; Wang Y. et al., 2021; Liu Z. et al., 2023), while platforms like AutoQSAR is used for model building (Yang et al., 2021; Arakal et al., 2021). Additionally, ML libraries such as R, Python (scikit-learn), and WEKA are crucial for statistical analysis and model development (Shah and Arumugam, 2024).

Khairullina and Martynova used GUSAR 2019 software, developed by the V.N. Orekhovich Institute, to perform QSAR analysis on derivatives of 5-ethyluridine, N2-guanine, and 6-oxopurine with anti-herpetic activity against Herpes simplex viruses (HSV) thymidine kinase. Twelve predictive models, based on quantitative neighborhoods of atoms (QNAs), multilevel neighborhoods of atoms (MNAs), and whole-molecule descriptors, demonstrated high accuracy in predicting pIC50 values. Virtual screening of the ChEMBL database using these QSAR models identified 42 new potential HSV-1 and HSV-2 TK inhibitors (compounds **#17-#58**) (Figure 12B) (Khairullina and Martynova, 2023). Similarly, Gaston-Mathé’s team evaluated a DL-based *de novo* design technology for generating lead compounds across 11 biological activity targets. Their QSAR models achieved precision between 0.67 and 1.0, and the AI algorithm generated 150 virtual compounds, with 11 synthesized compounds showing an 86% average success rate. One compound (compound **#59**) (Figure 12B) met all 11 objectives, while two met 10, highlighting the efficacy of combining AI-based *de novo* design with QSAR modeling for multi-parameter optimization (Perron et al., 2022). In the context of RNA-targeted drug discovery, Hargrove et al. developed QSAR models to predict binding parameters of small molecules to HIV-1 transactivation response (TAR) RNA. These models, built using multiple linear regression with feature selection, identified key properties influencing binding strength and kinetics, and were validated with new compounds, demonstrating their accuracy and broad applicability (Cai et al., 2022).

Recent advancements in AI techniques, particularly DL, along with the rapid expansion of molecular databases for virtual screening and significant improvements in computational power, have led to the emergence of a new field in QSAR applications, known as “deep QSAR” (Tropsha et al., 2024). One key advantage of “deep QSAR” over traditional methods is its ability to more effectively tackle multi-objective optimization tasks through knowledge transfer, leveraging diverse data sources to improve prediction accuracy across different tasks. For instance, Pérez-Castillo et al. developed a QSAR model based on DL strategies using hundreds of inhibitors of the SARS-CoV main protease (M^pro^) (Tejera et al., 2020). This model was used to virtually screen a large number of drugs from the DrugBank database, followed by docking and molecular dynamics analysis of the top 20 candidates. The results identified **Levothyroxine**, **Phenobarbital**, and **ABP-700** as the most promising inhibitors of the SARS-CoV-2 M^pro^ enzyme (Figure 12B). In a different approach, Ghosh et al. developed two supervised ML-based 3D-QSAR methods, CoMFA and CoMSIA, to analyze the structure-activity relationships of focal adhesion kinase inhibitors. Unlike 2D-QSAR, 3D-QSAR incorporates quantum chemical descriptors, molecular scaffolds, substitution constants, and various surface and volume descriptors, providing richer insights into the non-bonding interactions between receptors and ligands (Ghosh and Cho, 2023).

#### 5.2.2 Pharmacophore modeling

Pharmacophore modeling aims to identify the essential molecular features required for a ligand to interact with a biological target, typically a protein. These features include steric arrangements, electrostatic properties, aromatic rings, hydrogen bond donors and acceptors, and hydrophobic regions, which are abstracted into a pharmacophore—a 3D model representing the spatial arrangement of these key interactions necessary for binding and biological activity (van Drie, 2003). Pharmacophore modeling can identify key structural features shared by active ligands, which can then be used to screen for other molecules possessing these characteristics. To predict the activity of a new compound, QSAR models can be developed. While pharmacophore models highlight the essential features responsible for activity, QSAR provides a deeper understanding of how the chemical or physical properties of a ligand relate to its biological effect (Vyas et al., 2021). Pharmacophore models are useful for virtual screening, enabling the identification of new potential drug candidates by comparing large chemical libraries with the pharmacophore, thus accelerating hit identification, lead optimization, and drug design. The process typically involves identifying active compounds, extracting key molecular features, aligning them in 3D space, and generating a pharmacophore model to capture the essential interactions for biological activity (Lee et al., 2011). Tools like LigandScout, Discovery Studio, PharmMapper, and ZINCPharmer are commonly used to generate and evaluate pharmacophore models (Temml et al., 2014; Wang X. et al., 2017; Koes and Camacho, 2012).

Zhong et al. used MOE software to develop a pharmacophore model targeting Glucose Transporter 1 (GLUT1) and screened the National Cancer Institute (NCI) compound database, comprising 1,469 molecules. This screening identified 16 hit compounds, with four (compounds **#60-#63**) (Figure 12B) showing dose-dependent inhibition of glucose uptake and reduced colon cancer cell growth *in vitro*. Further results indicate that lead compound 57 was a GLUT inhibitor (Almahmoud et al., 2020). Similarly, Aulifa et al. performed an *in silico* study to evaluate the inhibitory activity of active compounds from the ashitaba plant compared to statins against the 3-hydroxy-3-methylglutaryl (HMG) Co-A reductase enzyme. Using pharmacophore modeling and docking simulations, 299 active HMG-CoA reductase inhibitors and 8,884 inactive compounds (decoys) were downloaded from the DUD-E database. Molecular docking revealed that 15 hit compounds exhibited low binding energy (∆G), suggesting potential inhibitory activity against HMG-CoA reductase. The lowest ∆G value was found in 3′-carboxymethyl-4,2′-dihydroxy-4′-methoxy chalcone (compound **#64**) (Figure 12B), which was lower than the ∆G value of the other comparator drugs, atorvastatin and simvastatin (Aulifa et al., 2024).

Traditional pharmacophore modeling relies on an effective pharmacophore with a protein-ligand eutectic structure as a reference, often depending on existing molecular libraries. However, obtaining these eutectic structures can be challenging, making *de novo* drug design difficult in many drug development projects (Giordano et al., 2022). To address this, Koes et al. proposed PharmRL, a novel approach that combines DL with geometric reinforcement learning to construct high-quality pharmacophores without requiring ligand information. Their algorithm demonstrated superior prospective virtual screening performance on the DUD-E dataset compared to randomly selecting ligand recognition features from eutectic structures. PharmRL was also tested on the COVID Moonshot dataset, effectively identifying potential lead molecules without fragment screening experiments (Aggarwal and D, 2024). Additionally, Imrie et al. introduced the DEVELOP model, which utilizes pharmacophore information to optimize precursor or lead compounds. By integrating the graph-based deep generative model DeLinker with a convolutional neural network, DEVELOP leverages 3D representations of molecules and target pharmacophores. Using the design of menin and mixed lineage leukemia (MLL) fusion protein inhibitors as a case study, they demonstrated that molecules generated by DEVELOP closely matched the input pharmacophore information (Imrie et al., 2021).

### 5.3 *De novo* drug design

*De novo* drug design involves creating novel molecules without relying on existing compounds or natural products. Traditional methods, such as fragment-based approaches, generate new molecules from scratch but often face challenges due to the complexity of molecular structures, making them difficult to synthesize (Schneider et al., 2017). Compared to virtual screening, drug design from scratch, aided by new algorithms for molecular design and evaluation, allows for more efficient exploration of a wider chemical space. While molecules proposed through *de novo* drug design are typically far from final drug candidates, they serve as valuable starting points for medicinal chemistry development (Tang et al., 2024). *De novo* drug design methods can be classified into structure-based and ligand-based approaches, depending on the level of molecular characterization (Schneider, 2014). Early structure-based methods involved growing ligands within a binding pocket’s steric and electronic constraints, either directly from protein structures or inferred from known ligand properties (Schneider and Fechner, 2005). However, these approaches often resulted in compounds with poor drug-likeness and synthetic feasibility, limiting their practical application. In contrast, recent advancements in ligand-based *de novo* drug design have shown promise in generating compound libraries that can be further analyzed using scoring functions to evaluate properties such as biological activity, synthetic accessibility, metabolism, and pharmacokinetics (Thomas et al., 2021; Atz et al., 2024).

**FIGURE 13:**
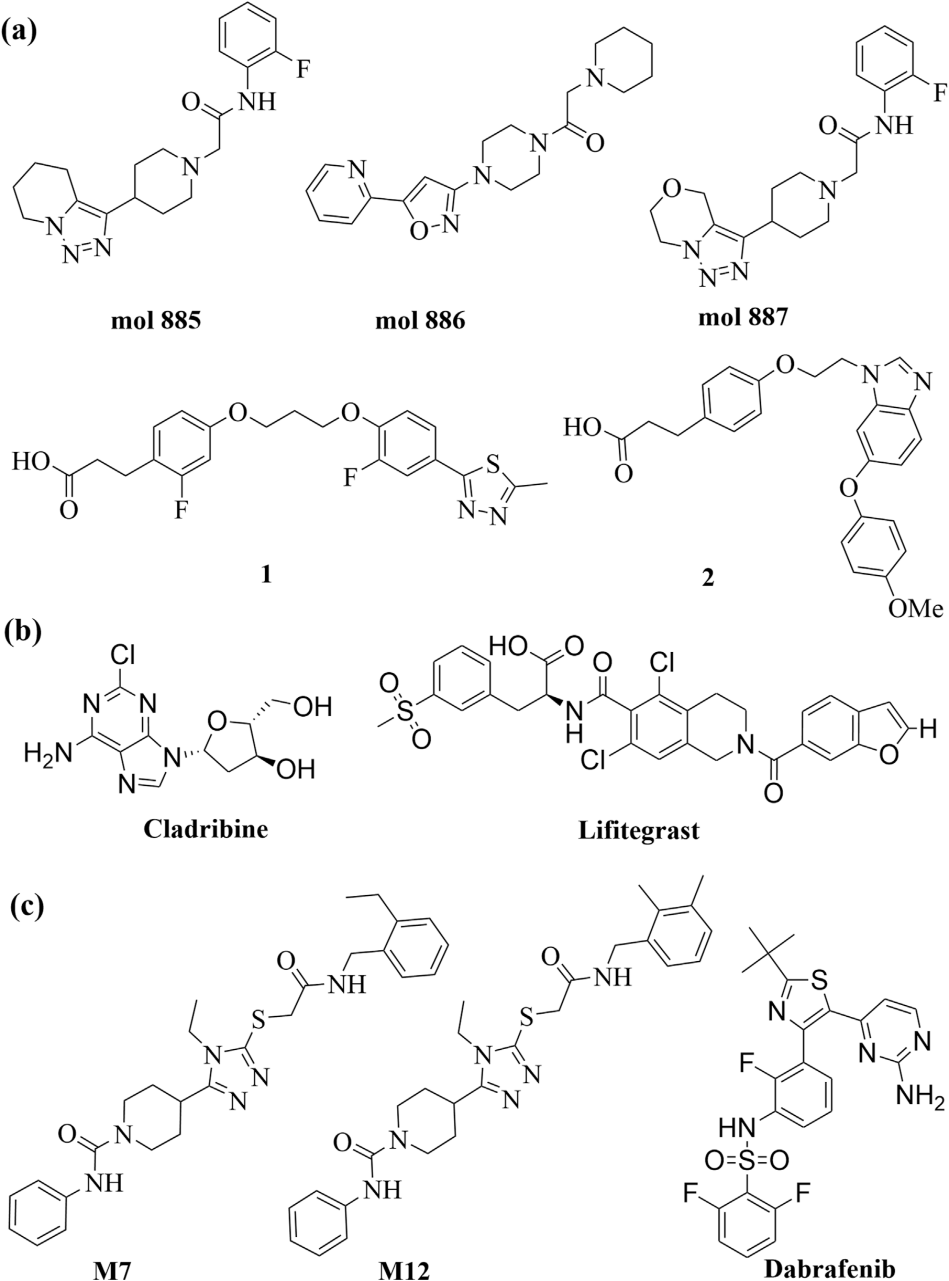
Chemical structures of small molecules identified through **(A)** *de novo* drug design, **(B)** drug repurposing, and **(C)** ADMET predictions.

In 2022, Gaston-Mathé et al. demonstrated the use of DL for multi-parameter optimization in ligand-based *de novo* drug design (Perron et al., 2022). They employed DL generative models to accelerate the identification of lead compounds that satisfied 11 different biological activity objectives. Using Servier’s project dataset, they established QSAR models for all 11 objectives, achieving moderate to high performance. A *de novo* algorithm based on DL was combined with the QSAR models to generate 150 virtual compounds expected to be active against all 11 objectives. Among the synthesized compounds, **mol 885** was active for all 11 indicators, while **mol 886** and **887** were active for 10, with the last indicator within the detection error range (Figure 13A). In 2024, Gisbert Schneider’s research team developed the DRAGONFLY computational method, which integrates graph neural networks and chemical language models (CLMs) to generate compound libraries with specific biological activity, synthesizability, and structural novelty (Atz et al., 2024). Using DRAGONFLY, they designed new ligands targeting the human peroxisome proliferator-activated receptor (PPAR) gamma subtype. Two top-scoring compounds (**1** and **2**) were synthesized and characterized through computational, biophysical, and biochemical methods (Figure 13A). The results identified potent PPAR partial agonists with favorable activity and selectivity profiles for both nuclear receptors and off-target interactions.

### 5.4 Drug repurposing

Drug repurposing, or the process of identifying new pharmacological effects for existing drugs, traditionally involved the labor-intensive task of analyzing vast amounts of medical literature and clinical data. This approach required interdisciplinary collaboration and was relatively inefficient. However, with the rapid expansion of bioinformatics and large-scale “omics” data, the time and cost associated with drug development have significantly decreased (Keiser et al., 2009). Computational drug repurposing has evolved from traditional methods based on chemical similarity and molecular docking to more advanced approaches that leverage systems biology for drug effect evaluation (Haupt and Schroeder, 2011; He et al., 2023). Recently, DL-based methods have shown great promise in automating the extraction of molecular structures, omics data, and clinical information, which improves the accuracy and interpretability of prediction models. These advances have accelerated drug repositioning. Several AI-driven platforms, such as DTINet, DEEPScreen, PandaOmics, PREDICT, SLAMS, and NetLapRLS, have been proposed to integrate heterogeneous data sources and identify potential drug repurposing opportunities (Luo et al., 2017; Rifaioglu et al., 2020; Liu B. H. M. et al., 2024; Gottlieb et al., 2011; Chen et al., 2021).

Zeng et al. proposed DTINet, a novel computational pipeline for predicting drug-target interactions (DTIs) by building a large-scale heterogeneous network that integrates data from various sources, including target genes, drugs, drug side effects, diseases, and other relevant factors (Luo et al., 2017). Focusing on the top 150 predictions, DTINet identified novel interactions between the drugs telmisartan, chlorpromazine, and alendronate, and cyclooxygenase proteins. These predictions were experimentally validated, revealing the potential of these drugs as cyclooxygenase inhibitors in preventing inflammatory diseases. In 2020, Doğan et al. introduced DEEPScreen, a DTI prediction system based on deep convolutional neural networks (CNNs), which uses 2-D structural compound representations to predict protein targets for drugs (Rifaioglu et al., 2020). As a case study, DEEPScreen predicted JAK proteins as new targets for **Cladribine**, and this was experimentally validated *in vitro* on cancer cells through STAT3 phosphorylation (Figure 13B). Meanwhile, Wang et al. employed PandaOmics, an AI-driven target discovery engine, to analyze large-scale transcriptomic data and identify novel therapeutic targets for endometriosis, including guanylate binding protein 2 (GBP2) and hematopoietic kinase (HCK), as well as the drug reuse target integrin β 2 (ITGB2). The FDA-approved drug **Lifitegrast**, a small molecule integrin antagonist for dry eye syndrome, was identified as a potential treatment for endometriosis (Figure 13B). *In vivo* experiments showed that Lifitegrast effectively inhibited lesion growth in a mouse model of endometriosis, suggesting its repurposing potential for this condition (Liu B. H. M. et al., 2024).

### 5.5 ADMET predictions

In addition to pharmacological efficacy, an ideal drug must also exhibit favorable ADMET (Absorption, Distribution, Metabolism, Excretion, and Toxicity) properties. Imbalances in these characteristics are a leading cause of late-stage drug failures and the withdrawal of approved drugs (Morgan et al., 2012). CADD plays a vital role in predicting ADMET properties, employing simulation techniques to forecast how drugs will behave in the body and their potential toxicity. Accurate ADMET predictions are essential in the early stages of drug discovery, guiding optimization efforts (Ferreira and Andricopulo, 2019). Recent advancements in AI and DL technologies have significantly enhanced the precision and efficiency of ADMET predictions. A new generation of predictors based on DL and big data promises to further streamline the drug discovery process, from laboratory studies to clinical applications (Keutzer et al., 2022; Alsultan et al., 2020). Popular tools for ADMET prediction include ADMETlab, ADMET Predictor, SwissADME, OptADMET, FAF-Drugs4, and Hit Dexter (Fu et al., 2024; Sohlenius-Sternbeck and Terelius, 2022; Daina et al., 2017; Yi et al., 2024; Lagorce et al., 2017; Stork et al., 2019).

In 2022, Iqbal et al. employed a comprehensive computational approach to identify potent never in mitosis A (NIMA)-related kinase 7 (NEK7) inhibitors, using both Autodock 4.2 and Molecular Operating Environment (MOE) 2015.10 for docking studies (Aziz et al., 2022). Their top compounds, **M7** and **M12**, demonstrated excellent binding affinities and were subsequently evaluated for ADMET properties using the ADMETlab 2.0 server (Figure 13C). Notably, both compounds showed superior human intestinal absorption (HIA) values compared to the standard HIV drug **Dabrafenib** (Figure 13C). In 2024, Cao et al. launched ADMETlab 3.0, an updated version of the web server that overcomes limitations of its predecessor by offering broader coverage, enhanced performance, improved API functionality, and better decision support (Fu et al., 2024).

### 5.6 Challenges of computer-aided drug design (CADD)

CADD has greatly enhanced drug discovery, but several challenges remain. SBVS requires high-quality 3D structures of biological targets, which are often unavailable, especially for membrane-bound or flexible proteins. This can lead to inaccurate docking results. To overcome this, advances in techniques like cryo-electron microscopy and homology modeling are being used to improve target structure prediction. LBVS faces limitations in predicting novel compounds, as it depends on known ligand-target interactions. Hybrid methods that combine SBVS and LBVS are now being explored to enhance screening accuracy and broaden the search for new drugs. *De novo* drug design offers the potential to generate entirely novel compounds, but the challenge lies in optimizing them for drug-like properties such as synthetic feasibility and biological activity. AI and ML are now being applied to refine *de novo* designs, increasing their success rate. Drug repurposing, although an efficient strategy for identifying new uses for existing drugs, often struggles with predicting the binding affinity of repurposed compounds to new targets. The use of large-scale molecular databases and advanced AI models can help in this area. ADMET predictions remain imprecise, complicating early-stage drug development. Enhanced computational models and multi-parameter optimization techniques are being developed to better predict these properties and identify safer drug candidates.

## 6 Conclusion and perspectives

This review highlights advanced strategies for the discovery and development of small-molecule drugs, delving into their underlying principles and examining their potential to the future of medicinal chemistry. As highlighted in this review, the integration of advanced techniques such as Click chemistry, TPD strategies, DELs technologies, and CADD has significantly improved the efficiency and effectiveness of drug discovery. Click Chemistry, with its rapid synthesis of diverse compound libraries, has reshaped how researchers approach the optimization of lead compounds. Its modular nature facilitates the incorporation of functional groups, allowing for the streamlined development of therapeutics tailored to specific targets. TPD technologies introduce a novel paradigm by utilizing the body’s natural degradation systems, enabling the targeting of previously undruggable proteins and expanding the therapeutic landscape. Similarly, DELs have transformed high-throughput screening by facilitating the parallel evaluation of millions of compounds, thereby accelerating the efficient identification of novel drug candidates. CADD represents a significant leap forward in the predictive capabilities of drug design, leveraging computational tools to refine candidate selection based on structural properties. This not only reduces the time and resources needed for experimental screening but also enhances the precision of drug design, aligning with the increasing demand for tailored therapies that meet individual patient needs.

Looking forward, the future of medicinal chemistry appears promising yet complex. The integration of AI into drug discovery processes is poised to further enhance the predictive capabilities and efficiency of these methodologies. However, challenges remain, particularly in terms of the biological validation of discovered compounds and the translation of preclinical findings into successful clinical applications. There is also a critical need to address the ethical implications and potential risks associated with these innovative approaches, ensuring that advancements are made responsibly. Additionally, by integrating multimodal data, including protein structure, sequence, and functional information, researchers can conduct a comprehensive analysis that provides deeper insights into drug design. This holistic approach enhances our understanding of molecular interactions and optimizes the development of targeted therapeutic drugs. Finally, to maximize the potential of these technologies, fostering interdisciplinary collaborations between chemists, biologists, pharmacologists, and data scientists will be essential. By combining expertise from various fields, the medicinal chemistry community can tackle the multifaceted challenges of drug discovery more effectively.

In conclusion, as we embrace these emerging strategies, there is a clear opportunity to inspire new research directions that not only advance our understanding of medicinal chemistry but also lead to the development of innovative therapeutic solutions. The integration of novel methodologies holds the potential to reshape the landscape of drug discovery, ultimately improving patient outcomes and addressing the pressing healthcare challenges of our time. By continuing to explore and refine these approaches, we can enhance our ability to combat a wide array of diseases, making significant strides towards more effective and targeted therapies.
